# Effects of acute Capsaicin analogue intake on athletic performance: a three-level meta-analysis

**DOI:** 10.1080/15502783.2026.2733576

**Published:** 2026-09-30

**Authors:** Zhu Huang, Hengzhi Deng, Shuiquan Tan

**Affiliations:** a School of Sports Science, Nanjing Normal University, Nanjing, People's Republic of China; b Faculty of Sports and Exercise Science, University of Malaya, Kuala Lumpur, Malaysia; c College of Physical Education, JiaYing University, Guangdong, People's Republic of China

**Keywords:** Capsaicin analogues, dose-response relationship, sports performance, three-level meta-analysis, TRPV1 agonist

## Abstract

**Background:**

Capsaicin analogues have been proposed as potential ergogenic aids, but their acute effects on athletic performance remain inconsistent. This study aimed to evaluate the effects of acute capsaicin analogue intake on exercise performance using a three-level meta-analysis.

**Methods:**

PubMed, Web of Science, Scopus, SPORTDiscus, and the Cochrane Library were searched up to April 1, 2025. Randomized controlled trials examining acute oral capsaicin analogue intake in healthy adults were included. Outcomes were classified into strength, power, muscular endurance, and aerobic capacity. Hedges’ g was calculated using a three-level random-effects model.

**Results:**

Twenty-one randomized crossover trials with 67 effect sizes were included. Acute capsaicin analogue intake significantly improved overall athletic performance compared with placebo (g = 0.20, 95% CI [0.07, 0.33], *p* < 0.01). Subgroup analyses showed significant effects on power (g = 0.39) and muscular endurance (g = 0.25), but not on strength or aerobic capacity. Phenylcapsaicin showed the largest effect, and benefits were more evident in trained participants. Dose-response analysis suggested a nonlinear pattern, with low-to-moderate doses showing more stable benefits.

**Conclusions:**

Acute capsaicin analogue intake may produce small but significant improvements in athletic performance, particularly in power and muscular endurance. However, due to heterogeneity, publication bias, and very low certainty of evidence, these findings should be interpreted cautiously.

## 1. Introduction

Capsaicin is the main bioactive compound in Capsicum plants. Due to its unique pharmacological properties, it has received widespread attention in recent years as a potential energy enhancer in the field of sports science [1,2]. The core mechanism of action of capsaicin stems from its specific activation of the Transient receptor potential vanilloid 1 (TRPV1) ion channel As a multimodal receptor widely distributed in sensory neurons and skeletal muscle cells [3], TRPV1 activation is closely related to the increased release of calcium ions (Ca^2^⁺) from the sarcoplasmic reticulum [4,5]. This change in intracellular calcium dynamics is thought to enhance the cross-bridge interaction between actin and myosin filaments, thus providing a reasonable molecular mechanism basis for capsaicin to enhance muscle contractility and power output [6,7].

Studies have shown that capsaicin can effectively reduce pain perception and rating of perceived exertion (RPE) in subjects during resistance training by inducing TRPV1 receptor desensitisation [8,9]. Capsaicin may exert potential ergogenic effects by reducing pain perception and perceived exertion, thereby improving exercise-performance outcomes such as muscular endurance, power output, strength, and endurance capacity. Currently, multiple randomised controlled trials (RCTs) have evaluated the effects of acute supplementation with capsaicinoids on different exercise-performance domains [10,11]. For example, in resistance exercise, acute ingestion of 12 mg of capsaicin 45 minutes before exercise has been reported to increase repetitions to failure in trained men [12]. However, the effects of capsaicin in endurance sports remain controversial. Human studies using low doses of commercially available products have shown that capsaicin did not significantly prolong time to exhaustion (TTE) or improved time trial performance [13]. Recent systematic reviews and preliminary meta-analyses have reported that capsaicinoids are robust in improving muscle endurance, but evidence for their effects on aerobic endurance remains unclear [2]. Specifically, capsaicin may have beneficial effects on muscular endurance and perceived fatigue after resistance exercise, but its effect on conventional aerobic time trials is not statistically significant [14,15]. Despite these promising findings, the wide range of supplement formulations (such as natural capsaicin versus synthetic phenylcapsaicin), dosage gradients (2–20 mg), timing of supplementation, and subject characteristics (such as training background) in the literature have led to significant inconsistencies in the results of various trials [16,17].

Given the increasing number of small-scale studies in this field and the high heterogeneity of their results, a comprehensive quantitative meta-analysis is needed to assess the overall impact of capsaicin on athletic performance and subjective fatigue. To fill this academic gap, this study employs a three-level meta-analysis model to systematically evaluate the overall effects of capsaicin on different athletic performances, and combines this with dose-response regression analysis to determine its potentially optimal supplementation strategy. The aim is to provide methodological and empirical references for sports science research and to offer evidence-based support for individualised and precise nutritional interventions for athletes.

## 2. Methods

This systematic review is reported in accordance with the Preferred Reporting Items for Systematic Reviews and Meta-analyses 2020 (PRISMA 2020) statement [18]. The complete list of PRISMA 2020 is provided in Appendix S1. In addition, this study has been registered on the international prospective systematic review registry platform (PROSPERO) with registration number CRD420261367687.

### 2.1. Eligibility criteria

For this systematic review and meta-analysis, the inclusion criteria were as follows: (1) randomised controlled trials in full-length peer-reviewed journals; (2) acute single oral administration of capsaicin; (3) exercise testing within 6 hours of administration; (4) original research articles; and (5) healthy adult participants (age ≥ 18 years). The exclusion criteria were: (1) studies involving co-administration with other substances; (2) animal or in vitro experiments; (3) studies that did not include exercise-related outcome measures; (4) studies on repeated or long-term capsaicin intake; (5) non-original research articles (e.g. clinical trial registration information, conference abstracts, review articles); (6) studies not written in English; and (7) studies with insufficient methodological information to validate the inclusion criteria. There were no restrictions on blinding status; both single-blind and double-blind randomised controlled trials were included to ensure a comprehensive synthesis of existing evidence. The inclusion criterion of exercise testing within 6 hours of oral capsaicin administration was adopted because studies showed that plasma capsaicin concentrations could return to baseline levels within 6 hours of administration.

### 2.2. Data sources and search

A systematic literature search was conducted in PubMed, Web of Science, Scopus, SPORTDiscus (via EBSCO), and the Cochrane Library to identify relevant studies published up to December 1, 2025. The following search strategy was applied: (“capsaicin” OR “8-methyl-*N*-vanillyl-6-nonenamide” OR “capsiate” OR “dihydrocapsaicin” OR “capsaicinoid” OR “nordihydrocapsaicin” OR “homodihydrocapsaicin” OR “homocapsaicin” OR “capsinoid”) AND (“exercise performance” OR “endurance” OR “aerobic capacity” OR “anaerobic capacity” OR “strength” OR “resistance training” OR “VO2max” OR “fatigue” OR “time to exhaustion”). Before the final analysis, the searches were updated on exact final search date using the same eligibility criteria and search strategy to identify newly published studies. The updated search identified the recently published study by [19], which met the predefined eligibility criteria and was therefore included in the systematic review and meta-analysis.

### 2.3. Data extraction

All retrieved records were imported into Microsoft Excel and EndNote (version 25) for management, and duplicates were identified and removed through manual cross-checking. Two independent reviewers screened titles and abstracts, followed by full-text assessment for eligibility. Any disagreements were resolved through discussion.

Data extraction was performed independently by the same reviewers using a standardised form. Extracted information included study design, participant characteristics (e.g. sample size, sex, and training status), capsaicin supplementation form (e.g. capsules or beverages), pre-exercise nutritional status, timing of supplementation, exercise protocols, and primary outcome measures (e.g. time to exhaustion, peak or mean power output, VO₂max or VO₂peak, muscular strength, vertical or countermovement jump height, and balance performance).

When necessary data were missing or unclear, attempts were made to contact the corresponding authors to obtain additional information.

### 2.4. Quality assessment

The methodological quality of the included studies was assessed using the Physiotherapy Evidence Database (PEDro) scale [20]. The PEDro scale consists of 11 items, which evaluate the methodological quality of randomised controlled trials from aspects such as inclusion criteria, randomisation, allocation concealment, similarity baseline, blinding, follow-up completeness, intention-to-concept analysis, and component comparison. One point is awarded for each item that is met. Since the first item is not included in the total score, the total score ranges from 0 to 10 points. A higher score indicates a higher methodological quality of the study.

The literature quality assessment was conducted independently by two researchers. In case of disagreement during the assessment process, the issues were first resolved through discussion and negotiation; if a consensus could not be reached, the final score was determined by reviewing the original text again and discussing it together.

### 2.5. Risk of bias assessment

The risk of bias in the included studies was assessed using the Cochrane Risk of Bias tool 2.0 (ROB 2) [21]. This tool systematically evaluates the risk of bias in randomised controlled trials in five areas: (1) bias in the randomisation process; (2) bias in deviation from the intended intervention; (3) bias due to missing outcome data; (4) bias in outcome measurement; and (5) bias in selective reporting of outcomes.

Each area was judged according to the signalling issues of ROB 2, and the risk of bias in each area and the overall risk was finally classified as “low risk”, “some concerns”, or “high risk”. The risk of bias assessment was completed independently by two researchers. If there were any disagreements during the assessment process, they were first resolved through discussion and negotiation; if no agreement could be reached, the final result was determined by reviewing the original text again and discussing it together.

### 2.6. Statistical analysis

#### 2.6.1. Data extraction, synthesis and effect measures

Data extraction was conducted independently by two researchers using a pre-designed data extraction table. The extracted data mainly included: first author, publication year, study design, subject characteristics, sample size, capsaicin and placebo supplementation regimen (dosage, form, and timing of supplementation), exercise testing protocol, and outcome data.

In this meta-analysis, “athletic performance” refers to standardised exercise-performance outcomes extracted from the included trials. These outcomes were grouped into four pre-specified physiological domains: strength, power, muscular endurance, and aerobic capacity. The overall athletic-performance effect therefore represents the pooled standardised effect across these exercise-performance domains rather than a single uniform performance test.

The data extraction process for this meta-analysis primarily focused on extracting and analysing exercise performance-related outcomes. Other physiological, biochemical, or subjective outcomes reported in the studies were not included in this meta-analysis.

All numerical outcome data included in the meta-analysis were available directly from the text, tables, or supplementary materials of the eligible studies. Consequently, WebPlotDigitizer was not used in this review, and no outcome data were subjectively estimated. If a study reported multiple related exercise outcome indicators, they were extracted according to pre-defined outcome priority rules to avoid duplicate data inclusion. For continuous outcome indicators, the effect size was expressed as the standardised mean difference (SMD) and its 95% confidence interval (95% CI) to unify the impact of inconsistencies in measurement units and testing methods across different studies.

#### 2.6.2. Meta-analysis and heterogeneity

All statistical analyses were performed in R software (v4.2.0; R Core Team, Vienna, Austria), and the meta and metafor packages were mainly used to implement the calculation process [22]. In the three-level framework, a random effects model was used to integrate the effect sizes to account for the possible heterogeneity among studies.

The random effects model assumes that the effect values ​​of different studies do not come from the same fixed population, but rather from a set of real effects with distribution characteristics. Considering that the included studies differ in terms of the characteristics of the subjects, the type of capsaicin supplementation, the dosage level, and the type of exercise performance test, the random effects model can more reasonably reflect the uncertainty and variability of the overall effect by introducing the effect distribution structure [23].

For data with nested structures, such as multiple performance indicators (e.g. muscle strength, explosive power, or VO2 max) reported in the same study, there is often statistical dependence among these effect sizes. If all of them are included in the analysis as independent observations, the independence assumption will be violated; while selecting only a single indicator may lead to insufficient information utilisation and reduced statistical efficiency. To address this issue, this study adopted the three-level meta-analysis method proposed by Assink and Wibbelink [24].

This method decomposes the total variance into three components: sampling error (level 1), within-study variation (level 2), and between-study variation (level 3), thus simultaneously considering the correlation between multiple effect sizes within the same study and the difference structure between different studies. This modelling approach improves the accuracy and statistical power of effect estimation while preserving complete data structure information. Model parameters are estimated using restricted maximum likelihood (REML) and supplemented by maximum likelihood (ML) sensitivity testing to verify the stability and consistency of the results [25]. All regression coefficients and their 95% confidence intervals are statistically inferred based on the t-distribution [26]. Simultaneously, the prediction interval (PI) is also calculated based on the t-distribution to describe the possible distribution range of the true effect, thereby supplementing the heterogeneity information that the 95% confidence interval cannot reflect. Regarding outlier identification, if the 95% confidence interval of a certain effect size does not overlap with the 95% confidence interval of the overall pooled effect, it is considered a potential outlier for further diagnosis. The leave-one-out analysis was then used to assess the impact of individual studies on the overall results.

Although heterogeneity can be measured by various statistical measures, including the Cochrane Q test, τ^2^, and I^2^ statistic, I^2^ is still used as the primary reporting measure due to its intuitiveness and wide applicability. This study interprets I^2^ according to previous literature standards: low heterogeneity (<25%), moderate heterogeneity (25%–50%), high heterogeneity (50%–75%), and high heterogeneity (>75%).

Furthermore, to avoid potential false negatives due to insufficient statistical power, this study performed a power analysis on the main pooled effects, which was conducted using the metameta software package to assess the overall statistical power [27].

#### 2.6.3. Moderators and subgroup analysis

To explore the potential sources and moderating factors of heterogeneity among studies, this study used a combination of subgroup analysis and meta-regression analysis to systematically evaluate categorical and continuous variables. According to methodological recommendations, each meta-regression analysis should include at least 10 studies, while each subgroup analysis should include at least 5 studies per group to ensure the stability and interpretability of the estimation results [28]. In addition, to reduce the risk of false negative results due to insufficient statistical power, this study calculated the statistical power of each subgroup effect.

Potential moderating variables included in the analysis were: (1) sex (male and mixed); (2) training status (trained and untrained); (3) capsaicin type (capsaicin, capsiate, phenylcapsaicin, dihydrocapsiate); Capsaicin is the major pungent capsaicinoid in chilli pepper and acts as a classical TRPV1 agonist [29]. Capsiate and dihydrocapsiate are non-pungent capsinoids found mainly in CH-19 Sweet pepper; they are structurally similar to capsaicin but contain an ester linkage rather than the amide linkage characteristic of capsaicinoids [30]. Phenylcapsaicin is a synthetic capsaicin analogue designed to retain capsaicin-like biological activity and is also considered a TRPV1 agonist [31]. Therefore, in the present review, capsaicin and phenylcapsaicin were classified as capsaicinoid-type compounds, whereas capsiate and dihydrocapsiate were classified as capsinoid-type compounds [32]. (4) athletic performance category (Aerobic capacity, Muscular Endurance, Strength, Power); and (5) blinded design. Participants were classified as trained when the original study described them as athletes, resistance-trained, endurance-trained, physically trained, or regularly engaged in structured exercise training. Participants were classified as untrained when the original study explicitly described them as sedentary, recreationally inactive, or not engaged in regular structured training.

To further improve the interpretability of the results and reduce the instability caused by the small sample size of a single indicator, this study integrates all performance indicators into four physiological functional domains based on the principles of exercise physiology and previous literature [33,34]. This classification is based on the main energy supply system, neuromuscular demand and task duration, as follows: (1) Strength: refers to the ability to generate maximum force output in a short time (≤10 seconds), which mainly depends on the ATP-creatine phosphate system. This category includes one maximum repetition (1RM), maximum voluntary contraction (MVC) or maximum isometric contraction (MVIC), peak torque and total torque of isokinetic muscle strength, as well as the maximum force output indicator in resistance training [35,36]. (2) Power: refers to the ability to generate power rapidly in a very short time, which also depends on the ATP-creatine phosphate system. This category includes the height of the countermovement jump (CMJ), short-distance sprint performance, and peak power or speed output in resistance or jumping tasks [37,38]. (3) Muscular endurance: refers to the ability to sustain repetitive muscle contractions under submaximal load until fatigue. This category includes total repetitions, volume load, time to exhaustion, and sustained performance during moderate-intensity resistance training (e.g. 60–70% 1RM) [39]. (4) Aerobic capacity: refers to the ability to sustain oxidative energy supply during prolonged exercise (>2–3 minutes). This category includes medium- and long-distance endurance performance, treadmill or bicycle endurance tests, VO₂max/VO₂peak related indicators, and physiological responses (e.g. heart rate) during prolonged endurance exercise [40,41]. Time-to-exhaustion outcomes were classified according to the testing protocol. TTE during resistance-type or localised muscular tasks was categorised as muscular endurance, whereas TTE during whole-body running or cycling protocols was categorised under aerobic-capacity outcomes.

All meta-regression analyses were performed using the metafor package, and the results were visualised using the ggplot2 and orchaRd packages. All statistical analyses were performed in the R software environment (R Foundation for Statistical Computing, Vienna, Austria) [42,43].

#### 2.6.4. Risk of publication bias and sensitivity analysis

To assess potential publication bias, this study used a contour-enhanced funnel plot combined with the Egger asymmetry test for analysis [44,45]. The Egger test was only performed when the number of included studies reached the statistically recommended standard (k ≥ 10) to improve the reliability of the test results [46]. The symmetry of the funnel plot was judged visually and combined with the results of the Egger test for comprehensive evaluation, thereby conducting qualitative and quantitative analysis of potential small sample effects and publication bias. When *p* > 0.05, it was considered that no significant risk of publication bias was detected.

Within a three-level meta-analysis framework, this study further conducted a multidimensional sensitivity analysis to systematically assess the robustness of the results regarding the effect of capsaicin supplementation on athletic performance. First, since most crossover design studies do not report paired correlation coefficients, different correlation coefficient scenarios (r = 0.2 and r = 0.8) were set in the main analysis to examine the impact of the correlation coefficient assumption on standard error estimation and pooled effect size.

Second, potential outliers and high-impact observations were identified using model diagnostic methods. Specifically, Cook distance, studentized residuals, and hat values ​​were used for evaluation at both the within-study (level 2) and between-study (level 3) levels [47,48]. Observations with potentially high-impact values ​​were identified when the Cook distance or hat value exceeded three times their mean, or when the absolute value of the studentized residual was greater than 3. Based on this, the model results before and after outlier treatment were compared to examine their impact on pooled effect size and heterogeneity estimation. Finally, a leave-one-out analysis was used to exclude individual studies one by one at the research level in order to assess the potential impact of individual studies on the overall effect estimate.

### 2.7. Certainty of the evidence

In interpreting the findings, this study employed the Grading of Recommendations Assessment, Development and Evaluation (GRADE) method to systematically assess the quality of evidence, taking into account the risk of bias and other uncertainties [49]. This method categorises the certainty of evidence into four levels: high, moderate, low, and very low. The GRADE assessment was completed independently by one researcher and reviewed by another to ensure objectivity and consistency in the assessment process.

Specifically, “high” quality evidence indicates that further research is unlikely to change the current effect estimate, and the conclusion has high credibility; “moderate” quality evidence suggests that further research may have a significant impact on the effect estimate and may change the current conclusion; “low” quality evidence means that the existing results have significant uncertainty, and further research is likely to change the effect estimate; while “very low” quality evidence indicates almost no confidence in the effect estimate, and the current results should be interpreted with caution.

## 3. Results

### 3.1. Research retrieved

In December 2025, a systematic literature search was conducted. A total of 1762 relevant records were initially identified using the PubMed (*n* = 239), Web of Science (*n* = 288), Cochrane Library (*n* = 3), Scopus (*n* = 800), Sport Discus (*n* = 430), and Scielo (*n* = 2) databases. Before screening, 253 duplicate articles were removed. After initial screening of the remaining 1509 articles by title and abstract, 1453 records that were not relevant to the research topic were excluded. Subsequently, the researchers conducted a rigorous quality assessment and feasibility analysis on the remaining 56 potentially eligible full-text reports. Based on the pre-defined inclusion and exclusion criteria, 35 reports were excluded for reasons including: incompatible intervention (*n* = 9), incompatible outcome measures (*n* = 16), incompatible study design (*n* = 9), and inability to obtain the full text (*n* = 1). Ultimately, all 21 included studies were crossover trials and were incorporated into this systematic review and meta-analysis. The detailed literature-search and study-selection process is shown in Figure 1.

**Figure 1.**
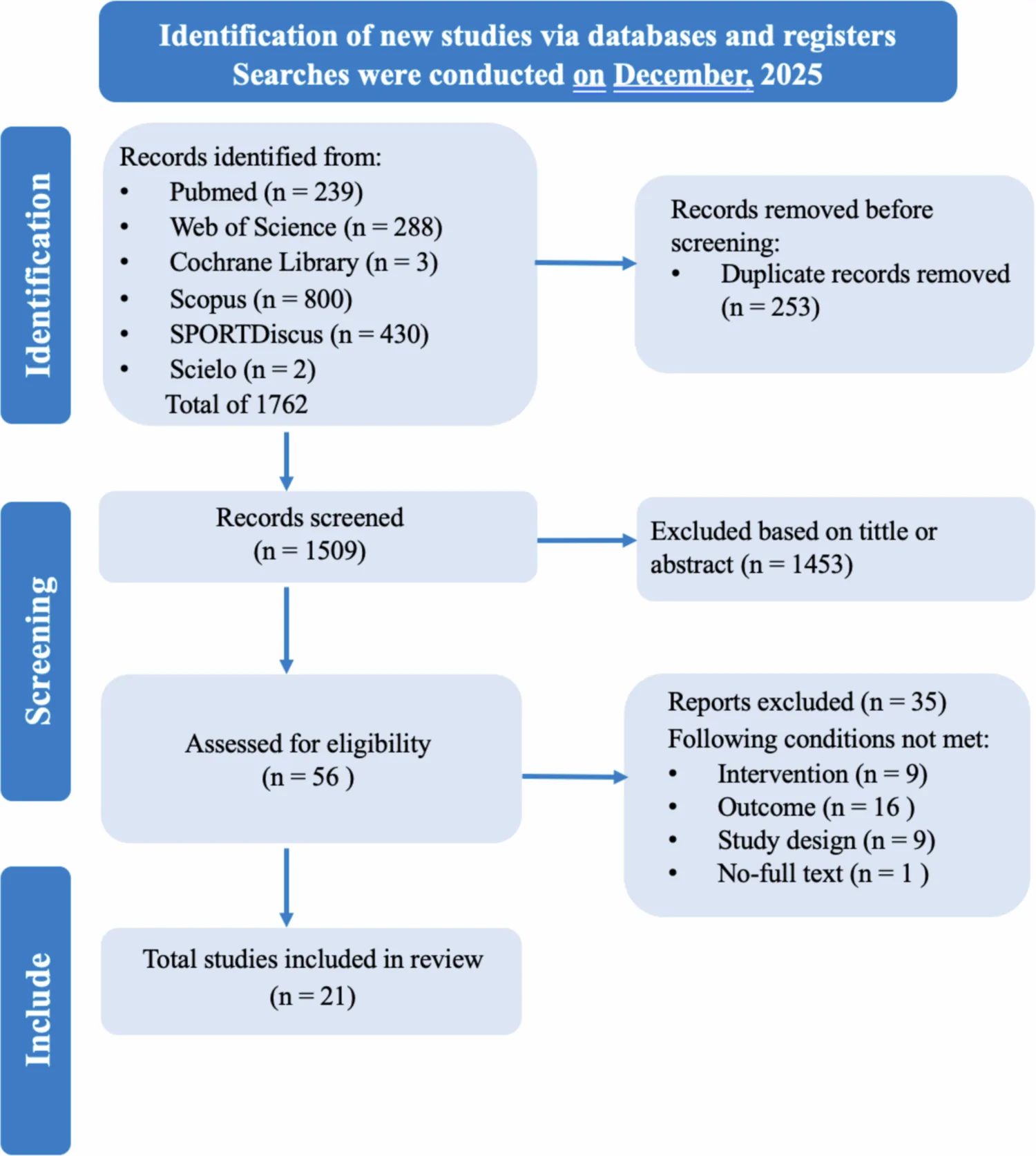
Literature screening flowchart.

### 3.2. Characteristics of included studies

#### 3.2.1. Characterisation of participants

Sample sizes ranged from 8 to 25 participants across all studies, totalling 348 participants. Of these 348 participants, 307 were male and 41 were female. Most of the included studies recruited only male participants (*n* = 17, k = 57); only four studies recruited both males and females (k = 10). Furthermore, most studies recruited trained participants (*n* = 18, k = 63), while a few recruited untrained participants (*n* = 3, k = 4), as detailed in Appendix S2.

#### 3.2.2. Exercise type

Based on the primary energy system or neuromuscular mechanism, movement types were classified, with 6 studies including Aerobic capacity (k = 10); 9 studies including Muscular Endurance (k = 19); 4 studies including Power (k = 13); and 7 studies including Strength (k = 25).

#### 3.2.3. Supplementation protocol and study design

Of the included studies, 19 (k = 64) supplemented capsaicin in capsule form, and 2 (k = 6) supplemented it in candy form. Of these, 15 studies used capsaicin, 3 used capsiate, 3 used phenylcapsaicin, and 1 used dihydrocapsaicin ester. Regarding dosage, all studies reported absolute doses ranging from 0.625 to 24 mg. Supplementation was primarily administered 45 minutes before exercise, with a few studies administering supplementation 30 minutes prior. All included studies were randomised crossover trials (4 were triple-blind, 15 were double-blind, and 2 were single-blind), detailed in Appendix S2.

#### 3.2.4. Main analysis

For overall athletic performance, the three-level variance analysis showed that capsaicin intake was significantly associated with a slight improvement in athletic performance (k = 67, g = 0.20, 95% CI [0.07, 0.33], *p* < 0.01) (see Figure 2). Furthermore, the heterogeneity analysis of the model indicated that 20.55% of the total variance was attributed to differences in effect size within the study (level 2), 34.17% to differences between studies (level 3), and the remaining 45.29% was explained by sampling error (level 1). Based on the three-level variance decomposition results, the combined proportion of between-study and within-study variance exceeded 50%, suggesting a certain degree of true heterogeneity in effect size. According to Hunter and Schmidt's criterion, when the proportion of variance explained by sampling error is less than 75%, significant true effect variance can be considered to exist in the data. Therefore, this study further conducted a moderating effect analysis to explore the sources of potential heterogeneity.

**Figure 2.**
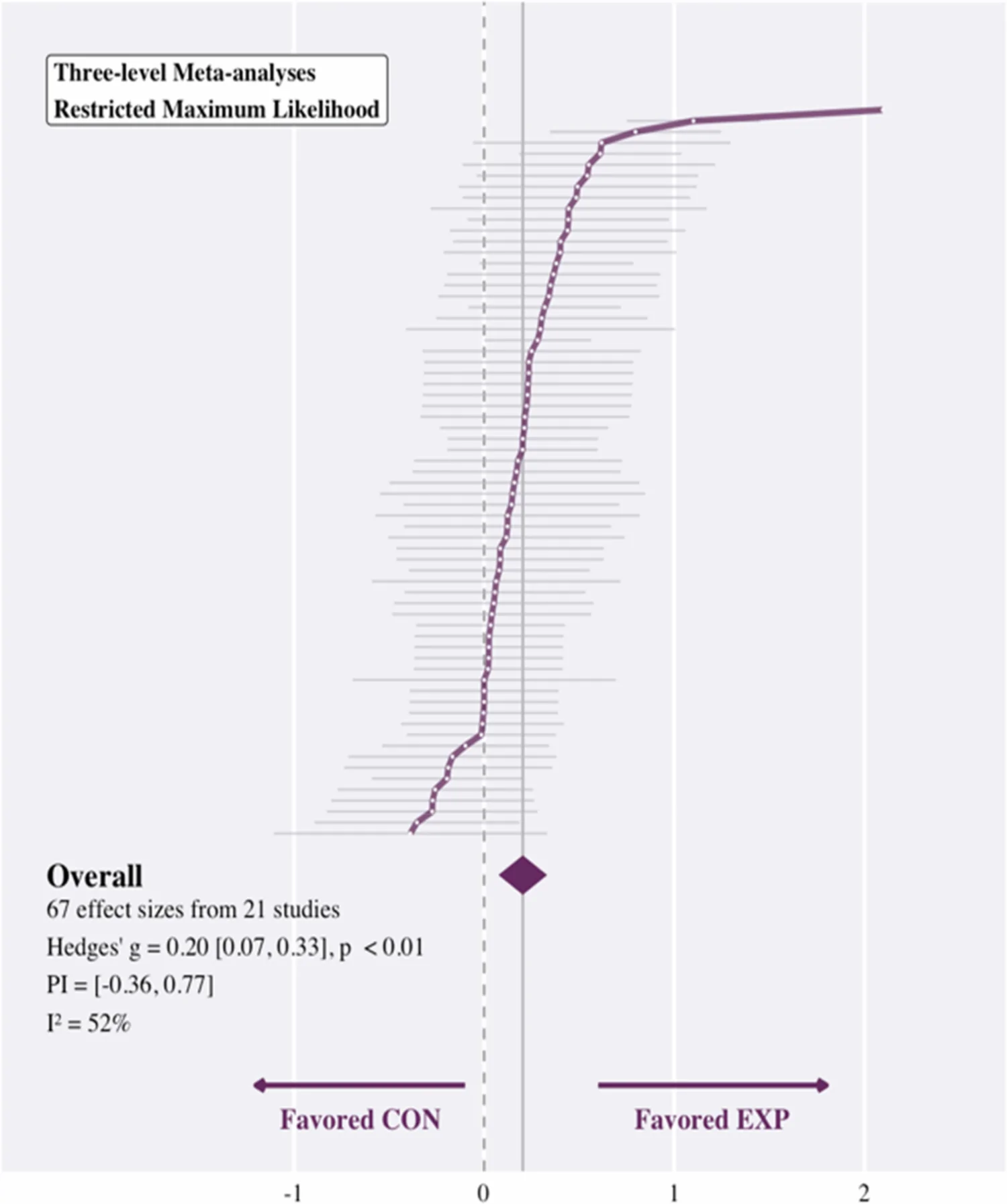
Forest plot showing the overall effect of capsaicin on athletic performance. Note: The diamond represents the pooled Hedges’ g, horizontal lines represent 95% confidence intervals, and PI indicates the prediction interval. *p* values indicate statistical significance, and I^2^ represents residual heterogeneity. CON = placebo/control group; EXP = capsaicin analogue supplementation group.

### 3.3. Moderator analysis

#### 3.3.1. Potential moderators of participants characterisation

Subgroup analysis showed that, in male participants, capsaicin analogue intake was associated with a significant improvement in pooled exercise-performance outcomes (g = 0.17, 95% CI [0.02, 0.31], *p* = 0.02, PI [−0.39, 0.72]), and a significant improvement was also observed in the mixed-sex sample (g = 0.36, 95% CI [0.07, 0.65], *p* = 0.02, PI [−0.25, 0.97]), as detailed in Figure 3.

**Figure 3.**
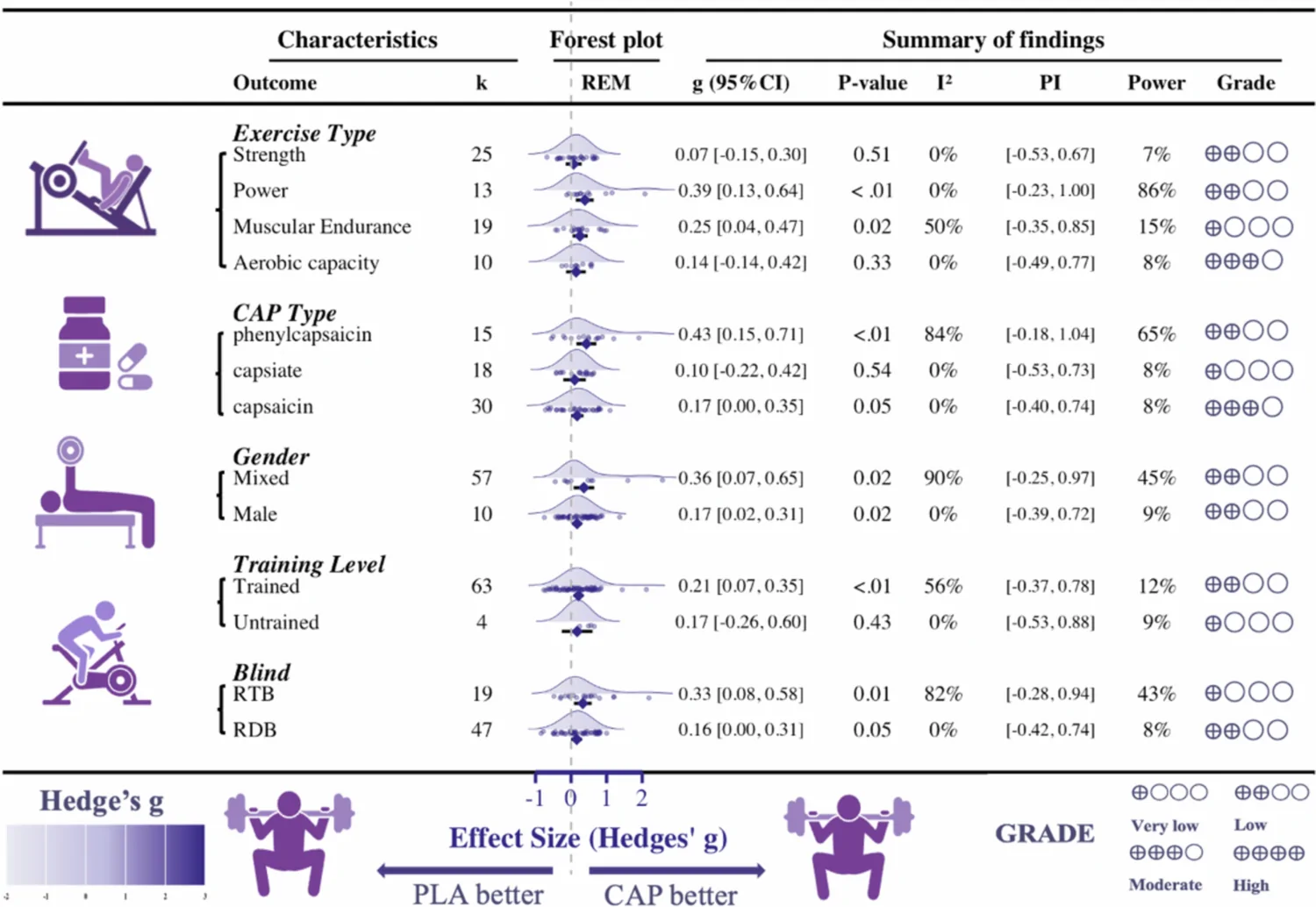
Summary of subgroup analyses of the effects of capsaicin on athletic performance. Note: k = number of effect sizes/comparisons; g = pooled Hedges’ g; 95% CI = 95% confidence interval; PI = prediction interval; I^2^ = residual heterogeneity; GRADE = Grading of Recommendations Assessment, Development and Evaluation; RDB = randomised double-blind; RTB = randomised triple-blind; REM = random-effects model.

Regarding training status, subgroup analysis indicated that capsaicin supplementation had a significant positive effect on trained participants (g = 0.21, 95% CI [0.07, 0.35], *p* < 0.001, PI [−0.37, 0.78]). In contrast, no statistically significant effect was observed in the untrained participants (g = 0.17, 95% CI [−0.26, 0.60], *p* = 0.43, PI [−0.53, 0.88]), see Figure 3 for details. One possible explanation is that trained individuals may be better able to translate capsaicin-related reductions in pain perception and perceived exertion into sustained performance during high-intensity exercise; however, this interpretation remains exploratory.

#### 3.3.2. Potential moderators of exercise type and capsaicin type

Subgroup analyses of different exercise types showed that capsaicin intake had varying effects on different exercise performance types. Specifically, capsaicin significantly promoted muscular endurance (g = 0.25, 95% CI [0.04, 0.47], *p* = 0.02, PI [−0.35, 0.85]) and also showed a significant trend towards improving power (g = 0.39, 95% CI [0.13, 0.64], *p* < 0.01, PI [−0.23, 1.00]). In contrast, no statistically significant effects were observed in strength (g = 0.07, *p* = 0.51) and aerobic capacity (g = 0.14, *p* = 0.33), as detailed in Figure 3.

In subgroup analyses with different supplementation forms, different capsaicin types exhibited different effect patterns. Among them, phenylcapsaicin showed the most significant promoting effect (g = 0.43, 95% CI [0.15, 0.71], *p* < 0.001, PI [−0.18, 1.04]). Capsaicin showed a marginally significant positive effect (g = 0.17, 95% CI [0.00, 0.35], *p* = 0.05, PI [−0.40, 0.74]), while the effect of the capsaicin analogue capsiate on athletic performance did not reach a statistically significant level (g = 0.10, *p* = 0.54), as detailed in Figure 3.

#### 3.3.3. Subgroup analysis by blinding design

Finally, in the subgroup analysis of the blinding methods, different levels of blinding had a certain impact on the effect size estimation. Studies using a randomised triple-blind (RTB) design showed a significant and relatively large intervention effect (g = 0.33, 95% CI [0.08, 0.58], *p* = 0.01, PI [−0.28, 0.94]). In contrast, studies using a randomised double-blind (RDB) design also showed a positive effect trend, but the effect size was smaller, and the statistical significance was at the critical level (g = 0.16, 95% CI [0.00, 0.31], *p* = 0.05, PI [−0.42, 0.74]), as detailed in Figure 3.

Overall, the effect size differed under different blinding quality conditions, but neither design showed a reversal of the effect direction, suggesting that the degree of blinding may affect the effect size estimation, but does not change the overall positive effect pattern of capsaicin on athletic performance.

#### 3.3.4. Potential moderating factors of supplement dosage

To investigate the dose-response relationship between capsaicin intake and improved athletic performance, this study conducted linear and nonlinear meta-regression analyses, as detailed in Figure 4.

**Figure 4.**
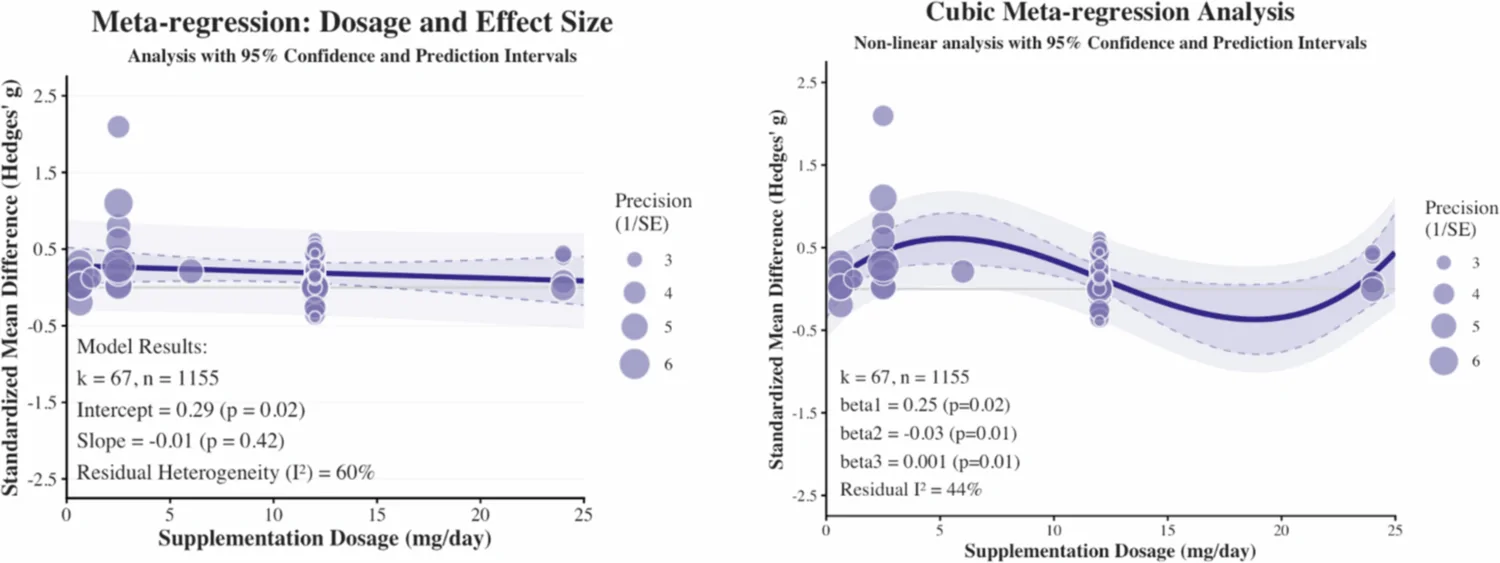
Meta-regression analysis of the relationship between capsaicin supplementation dose and effect size. Note: Each circle represents an individual effect size, with larger circles indicating greater precision, calculated as the inverse standard error (1/SE). The solid purple line represents the fitted meta-regression curve, the darker shaded area represents the 95% confidence interval, and the lighter shaded area represents the 95% prediction interval. The horizontal grey line indicates no effect (Hedges’ g = 0). In the linear meta-regression model, the intercept represents the estimated effect size when dosage equals zero according to the fitted linear model, and the slope represents the average change in Hedges’ g per 1-mg increase in dosage. In the polynomial meta-regression model, beta1, beta2, and beta3 represent the linear, quadratic, and cubic terms of the polynomial model, respectively. k = number of effect sizes; *n* = total sample size; I^2^ = residual heterogeneity.

Preliminary univariate linear meta-regression results showed that supplementation dose was not a significant linear predictor of effect size (slope = −0.0080, *p* = 0.419), and the model still exhibited high residual heterogeneity (residual I^2^ = 60%). However, the intercept term was statistically significant (intercept = 0.285, *p* = 0.020), suggesting that capsaicin supplementation may produce a positive effect at lower dose levels.

Given that the effects of bioactive substances in vivo typically exhibit nonlinear characteristics, a cubic polynomial meta-regression model was further used for fitting. The results showed that this model better characterises the dose-response relationship, with its linear term (β₁, *p* = 0.017), quadratic term (β₂, *p* = 0.01), and cubic term (β₃, *p* = 0.009) all reaching statistical significance. Meanwhile, the three-stage model reduced residual heterogeneity from 60% to 44.2%, suggesting that the nonlinear structure of the dose variable can explain some of the inter-study variability.

The dose-response curves predicted by the model exhibit a clear nonlinear pattern. In the lower dose range (approximately 2–10 mg/day), the effect size increases with increasing dose, showing a relatively stable benefit range. In the intermediate dose range (approximately 15–20 mg/day), the effect size changes tend to level off or even decrease slightly.

Although the model predicts a possible increase in effect size in the higher dose range (>25 mg/day), the uncertainty of the results is high due to the limited number of data points in this range and the significantly expanded prediction range, and should be interpreted with caution.

In summary, the effect of capsaicin on athletic performance is more likely to exhibit a nonlinear dose-response relationship than a simple linear increasing pattern. Current evidence suggests that a stable positive effect can be observed in the lower dose range, but the optimal dose range still needs further investigation to clarify.

### 3.4. Risk of bias and method quality

This study systematically evaluated the methodological quality of capsaicin-related studies using multiple tools. The PEDro scale results showed that most studies had high technical quality (scores concentrated between 8 and 10), indicating relatively standardised experimental designs in areas such as control setup, outcome reporting, and intergroup comparisons (see Appendix S5 for details). However, further evaluation using the more rigorous RoB 2.0 tool revealed that approximately 76% of the studies were classified as “some concerns” or “high risk” (Figure 5).

**Figure 5.**
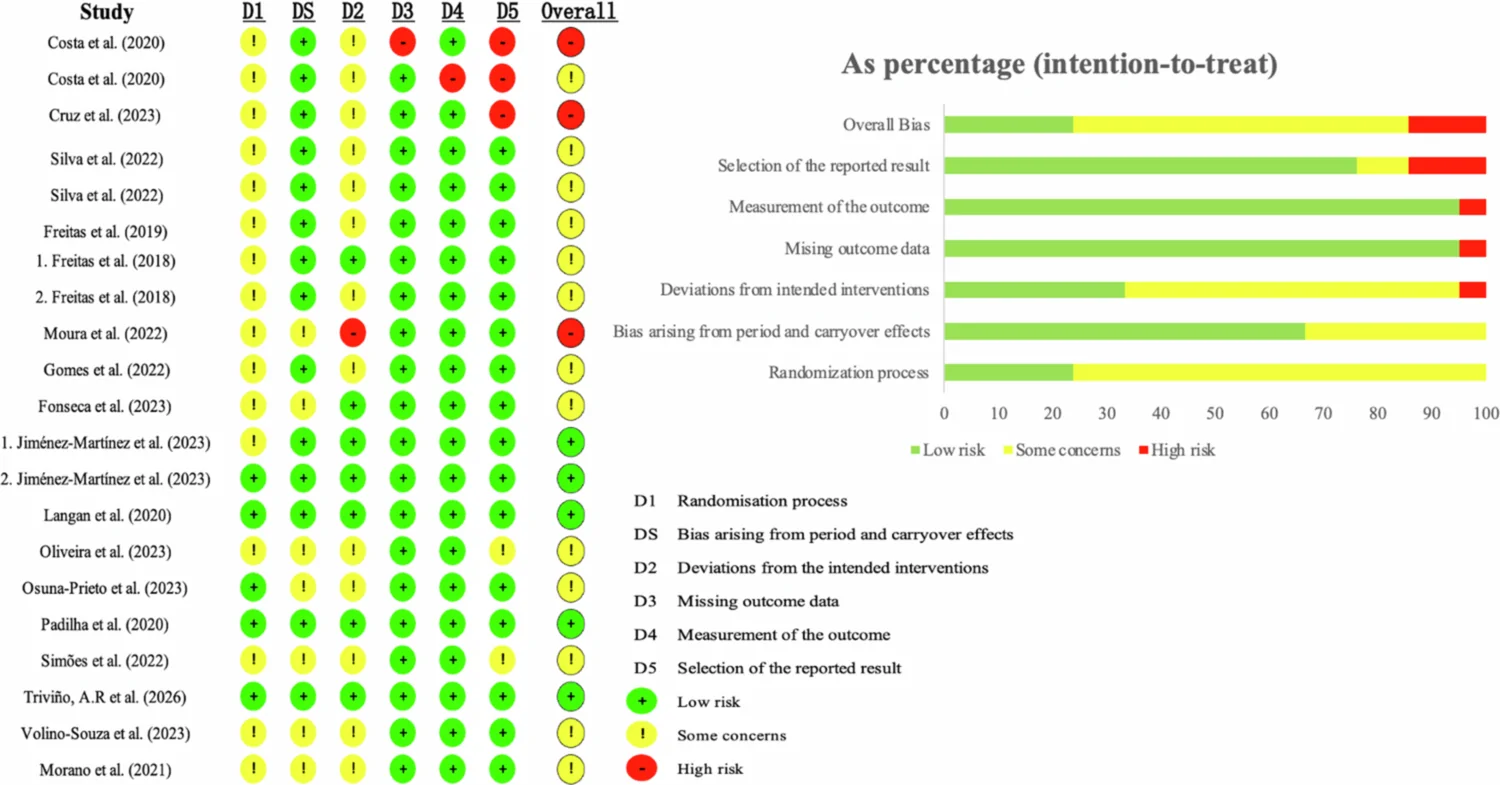
Summary chart of bias risk assessment in included studies.

Regarding internal validity, the RoB 2.0 results indicated that the main biases originated from the randomisation process (Domain 1) and selective outcome reporting (Domain 5). Some studies (e.g. Costa et al. [10];) did not adequately report the methods used to conceal the generation and allocation of random sequences, and were therefore classified as high-risk. This deficiency may lead to baseline imbalance, thus affecting the accuracy of effect estimates. Furthermore, researchers may tend to prioritise reporting significant results, thereby overestimating the true effect of capsaicin to some extent (see Figure 5).

Regarding publication bias, the funnel plot shows significant asymmetry, and the Egger test result is statistically significant (*p* < 0.01), indicating potential publication bias (Figure 5). The graphical distribution shows that studies with small samples and large effect sizes are mainly concentrated on the right side of the funnel plot, while the left side (studies with small samples and negative effects) is significantly lacking, reflecting a typical “drawer effect.” Subgroup analysis further shows that this bias exists in multiple strata, especially in studies related to the training population (Figure E) and explosive performance (Figure K) (see Appendix S3). The median statistical power of the study was only 11.4%, and the reproducibility index (R-index) was only 16.7%, This indicates limited reproducibility and a potentially high risk of false positive results. Therefore, it needs to be interpreted with caution. See Appendix S4 for details.

Based on the aforementioned risks of bias, inconsistencies, and precision issues, the GRADE system rated the overall quality of evidence as “Very Low.” The main reasons for the downgrade include: (1) significant risk of bias; (2) significant heterogeneity (I^2^ = 52.0%, and the prediction interval crosses zero); and (3) imprecision (most studies had small sample sizes, leading to unstable effect estimates). Although the quality of evidence improved slightly in some subgroups (such as phenylcapsaicin or aerobic capacity indicators), the overall certainty of the evidence remained low, as detailed in Appendix S6.

In summary, although capsaicin exhibits a certain positive effect on athletic performance, its conclusions are significantly influenced by methodological limitations and publication bias, and should be interpreted with caution. Future research should strengthen pre-registration systems, strictly implement randomisation and allocation concealment, and achieve transparent reporting of all outcomes to improve the reliability and reproducibility of evidence in this field.

### 3.5. Sensitivity analysis of the primary outcome

#### 3.5.1. Sensitivity analyses of the overall effect

This study conducted sensitivity analyses using different assumptions about the correlation coefficient r (r = 0.2 and r = 0.8) and different estimation methods to test the robustness of the pooled effect size.

Under the low correlation assumption (r = 0.2), the pooled effect size was g = 0.17 (95% CI [0.04, 0.31], *p* = 0.01), with heterogeneity I^2^ = 27% and statistical power of 0.08. Under the high correlation assumption (r = 0.8), the pooled effect size was g = 0.18 (95% CI [0.04, 0.32], *p* = 0.01), with I^2^ = 82% and statistical power of 0.18. Furthermore, under the ML model estimation, the pooled effect size remained stable (g = 0.21, 95% CI [0.08, 0.33], *p* = 0.002), corresponding to I^2^ = 51.70%.

Across sensitivity analyses using alternative within-participant correlation assumptions, different model estimation methods, and influence diagnostics, the pooled Hedges’ g ranged from 0.17 to 0.21, and the direction of the effect remained unchanged. Notably, the I^2^ varied significantly (27%–82%), indicating that the estimation of heterogeneity among studies is sensitive to the assumptions regarding paired correlation coefficients. However, this fluctuation did not affect the direction of the effect or the consistency of the statistical conclusions.

We identified potential outliers at the effect size level (level 2) and research level (level 3) using Cook distance, studentized residuals, and hat values, respectively, and refitted the three-level meta-analysis model based on the dataset after outlier removal. The diagnostic results identified several high-impact outliers (e.g. effect size ID 58: studentized residual = 5.51; ID 60: studentized residual = 3.35), and one study-level outlier (study ID 19: mean studentized residual = 3.03). After outlier removal, the model refit showed a pooled effect size of g = 0.15 (95% CI [0.07, 0.24], *p* < 0.01) and statistical power of 0.10. Notably, decreased numerically, with I^2^ decreasing to 0% and the prediction interval (PI) narrowing to [−0.04, 0.35]. Sensitivity analysis results showed that after excluding extreme influence points, the pooled effect size decreased slightly (from 0.20 to 0.15), but the statistical significance remained stable.

To assess the potential impact of individual studies on the pooled results, this study employed a leave-one-out analysis on the dataset that had been corrected based on a three-level model and aggregated into study-level effect sizes. The sensitivity analysis results show that after successively excluding any single study, the pooled effect size fluctuated within the range of g = 0.14–0.24, and all 95% confidence intervals did not cross zero, maintaining consistent statistical significance. Notably, after excluding [19], the pooled effect size decreased to g = 0.14 (95% CI [0.02, 0.25]), while the heterogeneity I^2^ decreased from approximately 45% to 0%. See Appendix S7 for details

#### 3.5.2. Sensitivity analysis of moderator effects

Sensitivity analyses were performed to examine whether moderator findings were influenced by individual studies. The subgroup estimates were recalculated after sequentially removing one study at a time. The direction and magnitude of most subgroup effects remained broadly similar, although some estimates became less precise in subgroups with few studies. These findings suggest that moderator results should be interpreted with consideration of subgroup sample size and precision.

Overall, the leave-one-out sensitivity analyses indicated that the direction and magnitude of most moderator estimates remained broadly similar after the sequential exclusion of individual studies. Excluding [19] reduced heterogeneity in some subgroup analyses but did not materially alter the direction of the corresponding effect estimates. Positive estimates were generally retained for trained participants, phenylcapsaicin, muscular endurance, and power outcomes. However, these findings should be interpreted cautiously because several subgroups were based on a limited number of studies or effect sizes and may therefore be sensitive to individual studies.

## 4. Discussion

This systematic review and three-level meta-analysis evaluated the acute effects of capsaicin and related capsaicinoids/capsinoids, including capsaicin, phenylcapsaicin, capsiate, and dihydrocapsiate, on exercise-performance outcomes. By applying a three-level model, this study accounted for the statistical dependence among multiple effect sizes extracted from the same study, thereby allowing a more appropriate synthesis of the available evidence than a conventional two-level approach. Overall, the findings suggest a small beneficial effect of acute capsaicin analogue intake on pooled exercise-performance outcomes, although the certainty of evidence remains limited by heterogeneity, small sample sizes, and variation in supplement type, dose, and exercise protocol.

### 4.1. Participant characteristics

The results showed that capsaicin analogue intake produced a significant positive effect in trained participants (g = 0.21, 95% CI [0.07, 0.35], *p* < 0.001), whereas no statistically significant benefit was observed in untrained participants (g = 0.17, 95% CI [−0.26, 0.60], *p* = 0.43). See Figure 3 for details. This difference may be due to training-induced changes in neuromuscular and metabolic adaptations, including improved motor unit recruitment efficiency, enhanced muscle oxidation capacity, and improved central fatigue perception regulation, thereby enhancing the responsiveness of exogenous nutritional intervention to exercise performance [50–53].

From a mechanistic perspective, capsaicin mainly promotes the release of sarcoplasmic reticulum calcium ions (Ca^2^⁺) by activating TRPV1 channels in skeletal muscle and sensory nerves, thereby enhancing the efficiency of excitation-contraction coupling and potentially improving muscle contraction performance and power output [4]. In trained individuals, long-term exercise load may induce expression regulation or enhanced functional sensitivity of TRPV1, resulting in a more significant calcium signal amplification effect under acute stimulation, thereby further amplifying the capsaicin-mediated intracellular Ca^2^⁺ response and excitation-contraction coupling effect [4]. In addition, people with training experience typically exhibit more efficient sarcoplasmic reticulum calcium ion (Ca^2^⁺) processing and reabsorption capabilities [6].

In untrained individuals, the absence of a clear effect may partly reflect greater variability in exercise tolerance and perceptual responses to capsaicin analogue intake; however, this interpretation should be considered exploratory because the untrained subgroup included relatively few effect sizes.

In terms of sex, subgroup analysis showed that capsaicin analogue intake was associated with a significant positive effect in male participants (g = 0.17, 95% CI [0.02, 0.31], *p* = 0.02, PI [−0.39, 0.72]) and in mixed-sex samples (g = 0.36, 95% CI [0.07, 0.65], *p* = 0.02, PI [−0.25, 0.97]; Figure 3). Although both subgroups showed statistically significant effects, the mixed-sex subgroup should be interpreted cautiously because the inclusion of both male and female participants may introduce additional biological variability. One possible explanation is that sex-related hormonal differences may influence capsaicin responsiveness through TRPV1-related mechanisms. Previous studies have shown that oestrogen can regulate TRPV1 expression or channel activity, which may alter sensitivity to capsaicin stimulation and modify the magnitude of downstream physiological responses [54,55]. In addition, sex differences in pain threshold, pain tolerance, and responses to capsaicin-induced burning sensations may further contribute to inter-individual variability [56]. Therefore, the current mixed-sex findings do not necessarily indicate a stronger effect in females or mixed samples; rather, they suggest that sex composition may be an important source of heterogeneity. Future studies should report sex-specific data and, where possible, control for menstrual-cycle phase or hormonal status to clarify whether the ergogenic effects of capsaicin analogues differ between males and females.

### 4.2. Type of exercise

The effects of acute capsaicin analogue intake differed across exercise-performance domains. The pooled analysis showed a significant small effect on muscular endurance (g = 0.25), whereas the effects on strength and aerobic capacity were not statistically significant. A significant pooled effect was also observed for power; however, this estimate was based on relatively few studies and was sensitive to individual influential studies.

For muscular endurance, the pooled finding was broadly consistent with individual resistance-exercise trials reporting improvements in repetitions completed, training volume, time to exhaustion, or resistance-exercise performance following acute capsaicin-related supplementation, including the studies by de Freitas et al., de Moura et al., and Jiménez-Martínez et al. [12,14,16]. Capsaicin may influence muscular-endurance performance through TRPV1-mediated modulation of pain perception and perceived exertion [4,7,36]. TRPV1 activation and subsequent functional desensitisation may increase tolerance to exercise-induced discomfort, while capsaicin-related changes in intracellular Ca^2^⁺ handling may theoretically help maintain excitation–contraction coupling during repeated muscular contractions [39]. However, these mechanisms were not directly examined in most of the included trials and should therefore be regarded as plausible explanations rather than established mediators of the pooled effect. After the exclusion of influential studies, the heterogeneity estimate decreased numerically and the direction of the muscular-endurance effect remained unchanged, indicating that the finding was not entirely dependent on a single study, although its precision remains limited by the number of available comparisons.

For power outcomes, individual studies assessed heterogeneous mechanical-performance tasks. For example, Jiménez-Martínez et al. [17] examined neuromuscular and mechanical performance following phenylcapsaicin intake. Jumping- and sprinting-based tasks differ in their biomechanical characteristics [57], energetic demands [58], and neuromuscular determinants [59]. These differences may have contributed to variability in the observed effects. Moreover, the sensitivity analysis indicated that the pooled power estimate was influenced by individual studies. Therefore, although the pooled effect was statistically significant, it should be interpreted cautiously.

For strength outcomes, no statistically significant pooled effect was observed, and the estimate remained nonsignificant across the sensitivity analyses. This finding was consistent with the study by Cross et al. [11], which did not identify a clear improvement in knee-extensor contractile function following low-dose capsaicin supplementation. Maximal strength is primarily determined by muscle morphology, cross-bridge force-generating capacity, and the recruitment and discharge rates of high-threshold motor units [60,61]. Acute modulation of pain perception may therefore be insufficient to produce a measurable improvement in maximal force production.

Findings for aerobic-capacity outcomes were also inconsistent across exercise protocols. Some running-based studies, including those by Costa et al. [10] and de Freitas et al. [62], reported improvements in time-trial performance or high-intensity intermittent time to exhaustion. Conversely, Langan and Grosicki reported no clear improvement in cycling time to exhaustion, while Morano et al. found no improvement in 10-km running performance. These contrasting findings suggest that the effects of capsaicin analogues may depend on the specific endurance protocol rather than applying uniformly to all aerobic tasks. Although capsaicin has been associated with increased lipid oxidation and possible glycogen-sparing effects [63,64], aerobic performance is strongly constrained by systemic oxygen delivery and cardiovascular capacity [65]. In addition, sensory or gastrointestinal discomfort may reduce exercise tolerance in some individuals; however, adverse effects were not systematically evaluated in the included studies. Consequently, no firm mechanistic conclusion can be drawn regarding the absence of a pooled aerobic-capacity effect.

### 4.3. Types of capsaicinoids

Phenylcapsaicin showed the largest pooled effect estimate among the compound-type subgroups. However, this finding was based on only three studies and should be considered exploratory. This difference is deeply rooted in the pharmacological properties of different compounds and their interaction with human receptors [4]. However, there is still limited evidence on whether its bioavailability is significantly improved compared to traditional capsaicin. The bioavailability of traditional natural capsaicin is often limited by the mucosal barrier and rapid metabolism when passing through the gastrointestinal tract [66]. Structurally modified capsaicin analogues such as phenylcapsaicin may have certain advantages in reducing spiciness. As a highly active compound with a Scoville rating, natural capsaicin can activate the TRPV1 receptor in the gastrointestinal tract at higher doses, thereby triggering a burning sensation and discomfort, which may limit its compliance and actual application dose in sports nutrition interventions [3,4,7]. Such discomfort may reduce tolerance, impair blinding, or distract participants during exercise testing, thereby offsetting potential performance benefits. In contrast, the lower spiciness of phenyl capsaicin may reduce sensory interference and improve athletes’ tolerance and compliance with intake in actual interventions [67,68], thus creating a different application advantage from natural capsaicin at the application level.

Capsiate failed to show statistically significant benefits in acute supplementation scenarios, a conclusion that remained robust across all sensitivity models. Existing studies have shown that while capsiate is also considered to activate TRPV1, its in vivo behaviour differs significantly from that of capsaicin. Studies have also shown that capsiate is readily hydrolysed in the gastrointestinal environment, and its metabolites are detected at low levels in the circulatory system, suggesting that its systemic bioavailability may be limited. For example, Snitker et al. [69] found in a randomised controlled study that the detectable circulating levels of dihydrocapsiate after oral administration were low, and its metabolism was rapid, which may limit its sustained effects in peripheral tissues (such as skeletal muscle) [70]. Furthermore, studies have indicated that the physiological effects of capsiate are more manifested through energy metabolism regulation mediated by sensory nerve pathways, rather than directly enhancing neuromuscular functional output [69]. Therefore, in acute supplementation scenarios, its low systemic exposure level and its likely metabolically modulating mechanism of action may together contribute to its difficulty in producing a detectable improvement in motor performance in a short period of time.

### 4.4. Supplementary strategies

In exercise intervention studies of capsaicin, the choice of dosage does not linearly increase the effect, but rather there is a complex, possibly inverted U-shaped window period. Data from this meta-analysis show that in the lower dose range, exercise performance improves with increasing dose, but after reaching a certain threshold, this gain begins to plateau or even show negative growth. Current academic consensus tends to regard 12 mg as the ideal single supplemental dose for adult male subjects [2,17,71]. Multiple experiments by de Freitas et al. have demonstrated that 12 mg of capsaicin taken 45 minutes before exercise can significantly increase the total number of repetitions (NRM) and total lifting load in squat exercises [2,12]. Tests on 1500-metre and 3000-metre runs showed that 12 mg of capsaicin can shorten running time by about 5-20 seconds [62,72]. This effect is considered to have reached the “effective pressure” of sympathetic nerve activation, making the fat oxidation and glycogen saving effects statistically significant in practice [73].

In stark contrast to 12 mg, supplementation regimens such as 1.2 mg or lower were deemed ineffective in most studies [13]. Although 1.2 mg had a weak effect on resting metabolism, this intensity of TRPV1 stimulation was insufficient for trained athletes to overcome the strong physiological noise generated by exercise itself. It could not induce sufficient catecholamine levels to drive substrate switching, nor could it produce an effective desensitising effect on widely activated type III/IV fibres [13,16].

Evidence concerning doses above 25 mg remains sparse.Opheim and Rankin's study pointed out that a high dose of 25.8 mg caused widespread gastrointestinal heartburn, nausea and diarrhoea. From a physiological perspective, this intense gastrointestinal discomfort can have a new effect, potentially counteracting the analgesic effects of capsaicin and distracting athletes, thus leading to a decline in athletic performance [73–75]. Extremely high-intensity TRPV1 activation can completely disrupt the phosphorylation state of channel proteins, causing them to enter a long-term unresponsive state. This means that the receptor system, which should assist muscle contraction, may be paralysed, thereby interfering with the normal mechanical feedback of the muscle [76]. Although gastrointestinal symptoms have been reported in some contexts, adverse effects were not systematically evaluated in the included trials. Therefore, no definitive conclusion can be drawn regarding the efficacy, tolerability, or optimal upper dose of acute capsaicin analogue supplementation.

### 4.5. Quality of blinding

Because capsaicin has a strong spiciness and heat sensation, subjects are very likely to find out whether they are in the experimental group or the control group through sensory feedback, which can lead to a serious “blinding” phenomenon. In the subgroup analysis of this study, the difference in the blinding scheme had a significant impact on the conclusion that capsaicin improves athletic performance. The results showed that the experiment with the triple-blind design observed a more prominent exercise gain effect compared with the traditional double-blind design. This difference first reflects the core position of the rigour of the study design in the evaluation of sports supplements. In the triple-blind design, neither the data analyst nor the experimenter knew the group information. Through multiple isolations, the potential interference of researcher bias and expected effects on the experimental results was effectively eliminated, so that the biological effects of capsaicin could be more purely manifested [77]. This rigorous control may reveal the true effect that was masked by noise in the past studies with looser designs.

From a physiological perspective, the risk of capsaicin “blinding” is key to discussing the rigour of its design. Due to the strong spiciness and potential gastrointestinal burning sensation of capsaicin, traditional double-blind designs often fail to completely mask the sensory differences between the intervention and the placebo [78]. If subjects can identify the intervention through taste or sensation, they may experience a strong psychological suggestion, thereby reducing perceived exertion (RPE) through central nervous system regulation. However, the triple-blind design in this study still showed a more robust positive effect, suggesting that the improvement of athletic performance by capsaicin is not simply due to the psychological placebo effect, but has a deep biological basis.

Capsaicin primarily exerts its effects by activating TRPV1 receptors. TRPV1 is widely distributed in sensory neurons and skeletal muscle. Upon activation, it promotes the release of calcium ions (Ca^2^⁺) in the sarcoplasmic reticulum, enhances the contractility of myofibrils, and may alleviate exercise-induced pain by regulating neurotransmitter release [79]. In addition, capsaicin is also thought to promote catecholamine secretion by activating the sympathetic nervous system, thereby accelerating lipid oxidation and conserving muscle glycogen [80]. The high heterogeneity observed in triple-blind studies may be due to individual differences in the sensitivity of subjects to capsaicin and the different degrees of calcium ion balance dependence of different types of exercise [81]. In conclusion, although a rigorous blinding protocol can more clearly capture the effects of capsaicin, future research should focus on sensory desensitisation of subjects and the development of more effective placebo analogues to confirm its physiological gain pathway.

### 4.6. Advantages and limitations

The main advantage of this study lies in its use of a three-level meta-analysis model, which effectively addresses the dependence of multiple effect sizes in a single study, thereby improving the accuracy of effect estimation. Furthermore, multidimensional sensitivity analysis and nonlinear modelling further enhance the robustness of the results.

However, this study also has certain limitations. First, [19] significantly influenced heterogeneity in some subgroups, suggesting that individual study results may exhibit high variability. Second, the low proportion of female participants limited the extrapolation of gender-related conclusions. Additionally, the relatively insufficient data in the high-dose range resulted in higher uncertainty in the dose-response curve within this range.

This lower-dose efficacy might be confounded by the inclusion of specific derivatives, particularly phenylcapsaicin. Emerging evidence suggests that phenylcapsaicin exhibits higher bioavailability and may be more active at significantly lower dosages compared to natural capsaicin extracts. Therefore, the overall trend observed in our regression model may not universally apply to all capsaicin forms. Future high-quality trials directly comparing the dose-response relationships of different capsaicin derivatives are warranted to isolate these specific effects.

### 4.7. Practical significance and future direction

Based on the dose range examined in the included studies, acute capsaicin analogue supplementation was generally administered within approximately 0.625–24 mg, most commonly 30–45 minutes before exercise. The present findings suggest that lower-dose strategies, particularly within the range of approximately 2–12 mg, may be practically relevant for resistance-type muscular endurance and power-related tasks. However, because compound type, dose, and exercise modality were not fully separable across studies, this range should be considered a tentative practical reference rather than a definitive dosing recommendation.

Future research should focus on: (1) the effects of capsaicin on central nervous system drive; (2) its physiological response under high temperature or high load conditions; and (3) precision nutrition strategies based on individual characteristics (such as sex and training level).

## 5. Conclusions

This three-level meta-analysis indicates that acute capsaicin analogue intake is associated with a small improvement in pooled exercise-performance outcomes. Significant benefits were observed for power and muscular endurance, whereas no statistically significant effects were detected for strength or aerobic capacity. Although trained participants showed a significant pooled effect and phenylcapsaicin produced the largest estimate among the compound-type subgroups, these findings should be considered exploratory because the corresponding subgroups included relatively few studies and the different capsaicin-related compounds cannot be assumed to be dose-equivalent.

The dose–response analysis suggested a nonlinear pattern, with low-to-moderate doses showing relatively more stable beneficial effects. However, this pattern should not be interpreted as establishing an optimal dose because compound type, absolute dose, and exercise modality could not be fully separated, and evidence at higher doses was sparse. Overall, acute capsaicin analogue supplementation may provide a modest ergogenic benefit, particularly for resistance-type muscular endurance and power-related tasks. Nevertheless, confidence in these findings is limited by between-study heterogeneity, small sample sizes, the predominance of male and trained participants, potential publication bias, and the very low certainty of the available evidence. Future adequately powered trials should directly compare different capsaicin-related compounds and doses, include more female and untrained participants, and systematically evaluate tolerability and adverse effects.

## Supplementary Material

Supplementary Material Electronic_Supplementary.docx

## References

[1] O’Neill J , Brock C , Olesen AE , et al. Unravelling the mystery of capsaicin: a tool to understand and treat pain. Pharmacol Rev. 2012;64(4):939–971. doi: 10.1124/pr.112.006163 PMC3462993 23023032

[2] Grgic J , Memon AR , Chen S , et al. Effects of capsaicin and capsiate on endurance performance: a meta-analysis. Nutrients. 2022;14(21):4531. doi: 10.3390/nu14214531 PMC9655695 36364793

[3] Caterina MJ , Schumacher MA , Tominaga M , et al. The capsaicin receptor: a heat-activated ion channel in the pain pathway. Natur. 1997;389(6653):816–824. doi: 10.1038/39807 9349813

[4] Caterina MJ , Julius D . The vanilloid receptor: a molecular gateway to the pain pathway. Annu Rev Neurosci. 2001;24:487–517. doi: 10.1146/annurev.neuro.24.1.487 11283319

[5] Clapham DE . TRP channels as cellular sensors. Natur. 2003;426(6966):517–524. 10.1038/nature02196 14654832

[6] Fitts RH . The cross-bridge cycle and skeletal muscle fatigue. J Appl Physiol (1985). 2008;104(2):551–558. doi: 10.1152/japplphysiol.01200.2007 18162480

[7] Allen DG , Lamb GD , Westerblad H . Skeletal muscle fatigue: cellular mechanisms. Physiol Rev. 2008;88(1):287–332. doi: 10.1152/physrev.00015.2007 18195089

[8] Kelava L , Nemeth D , Hegyi P , et al. Dietary supplementation of TRPV1 agonists reduces serum cholesterol: a meta-analysis. Crit Rev Food Sci Nutr. 2022;62(25):7025–7035. doi: 10.1080/10408398.2021.1910138 33840333

[9] Quizzini GH , Campos JMS , Nunes FAS , et al. Potential ergogenic aid of capsaicinoids or capsinoids: meta-analysis. Nutr Res. 2026;146:13–25. doi: 10.1016/j.nutres.2025.12.004 41505853

[10] Costa LA , Freitas MC , Cholewa JM , et al. Acute capsaicin analog supplementation improves 400 M and 3000 M running time-trial performance. Int J Exerc Sci. 2020;13(2):755–765. doi: 10.70252/FQFA1206 PMC7241615 32509117

[11] Cross BL , Parker D , Langan SP , et al. Effect of a commercially available low-dose capsaicin supplement on knee extensor contractile function. Int J Exerc Sci. 2020;13(2):312–318. doi: 10.70252/ELCT8391 PMC7039477 32148619

[12] de Freitas MC , Cholewa JM , Freire RV , et al. Acute capsaicin supplementation improves resistance training performance in trained men. J Strength Cond Res. 2018;32(8):2227–2232. doi: 10.1519/JSC.0000000000002109 28682933

[13] Langan SP , Grosicki GJ . Commercially available capsaicin supplement fails to enhance time-to-exhaustion during cycling. Int J Exerc Sci. 2020;13(2):225–233. doi: 10.70252/FLPN8768 PMC7039493 32148618

[14] de Moura HPSN , Esteves GP , de Souza Neto AV , et al. Acute low-dose capsiate supplementation improves upper body resistance exercise performance in trained men: a randomized, crossover and double-blind study. Int J Exerc Sci. 2022;15(2):1007–1018. 10.70252/YNEM9216 PMC9458288 36159159

[15] Morano AEVA , Padilha CS , Soares VAM , et al. Capsaicin analogue supplementation does not improve 10 km running time-trial performance in Male amateur athletes: a randomized, crossover, double-blind and placebo-controlled study. Nutrients. 2021;13(1):34. doi: 10.3390/nu13010034 PMC7823530 33374147

[16] Jiménez-Martínez P , Cornejo-Daza PJ , Sánchez-Valdepeñas J , et al. Effects of different phenylcapsaicin doses on resistance training performance, muscle damage, protein breakdown, metabolic response, ratings of perceived exertion, and recovery: a randomized, triple-blinded, placebo-controlled, crossover trial. J Int Soc Sports Nutr. 2023;20(1):2204083. doi: 10.1080/15502783.2023.2204083 PMC10124973 37086038

[17] Jiménez-Martínez P , Sánchez-Valdepeñas J , Cornejo-Daza PJ , et al. Effects of different phenylcapsaicin doses on neuromuscular activity and mechanical performance in trained Male subjects: a randomized, triple-blinded, crossover, placebo-controlled trial. Front Physiol. 2023;14:1215644. doi: 10.3389/fphys.2023.1215644 PMC10433207 37601635

[18] Page MJ , McKenzie JE , Bossuyt PM , et al. PRISMA 2020 statement. Brit Med J. 2021;372:n71. doi: 10.1136/bmj.n71 PMC8005924 33782057

[19] Triviño AR , Díaz-Romero C , Martin-Olmedo JJ , et al. Acute phenylcapsaicin supplementation improves CrossFit® performance: a randomized, triple-blind, placebo-controlled crossover trial. Int Soc Sports Nutr. 2026;23(1):2615274. doi: 10.1080/15502783.2026.2615274 PMC12810409 41536004

[20] de Morton NA . PEDro scale validity. Aust J Physiother. 2009;55(2):129–133. 10.1016/s0004-9514(09)70043-1 19463084

[21] Sterne JAC , Hernán MA , Reeves BC , et al. ROBINS-I tool. Brit Med J. 2016;355:i4919. doi: 10.1136/bmj.i4919 PMC5062054 27733354

[22] Viechtbauer W . Metafor package. J Stat Softw. 2010;36(3):1–48.

[23] Borenstein M , Hedges LV , Higgins JPT , et al. Fixed vs random effects. Res Synth Methods. 2010;1(2):97–111. 10.1002/jrsm.12 26061376

[24] Assink M , Wibbelink CJM . Three-level meta-analysis. TQMP. 2016;12:154–174.

[25] Patterson HD , Thompson R . Inter-block information recovery. Biometrika. 1971;58(3):545–554. doi: 10.1093/biomet/58.3.545

[26] Jukic I , Pérez Castilla A , García Ramos A , et al. Velocity loss thresholds review. Sports Med. 2023;53(1):177–214. doi: 10.1007/s40279-022-01754-4 PMC9807551 36178597

[27] Quintana DS . Statistical power in meta-analysis. AMPPS. 2023;6(1):25152459221147260. doi: 10.1177/25152459221147260

[28] Lauersen JB , Bertelsen DM , Andersen LB . The effectiveness of exercise interventions to prevent sports injuries: a systematic review and meta-analysis of randomised controlled trials. Br J Sports Med. 2014;48(11):871–877. doi: 10.1136/bjsports-2013-092538 24100287

[29] Iida T , Moriyama T , Kobata K , et al. TRPV1 activation and induction of nociceptive response by a non-pungent capsaicin-like compound, capsiate. Neuropharmacology. 2003;44(7):958–967. doi: 10.1016/S0028-3908(03)00100-X 12726827

[30] Paulsen TR , Stiller S , Weber K , et al. A 90-day toxicity and genotoxicity study with high-purity phenylcapsaicin. Toxicol Res Appl. 2018;2:1–15. doi: 10.1177/2397847318773060

[31] Reyes-Escogido ML , Gonzalez-Mondragon EG , Vazquez-Tzompantzi E . Chemical and pharmacological aspects of capsaicin. Molecules. 2011;16(2):1253–1270. doi: 10.3390/molecules16021253 PMC6259610 21278678

[32] Sasahara I , Furuhata Y , Iwasaki Y , et al. Assessment of the biological similarity of three capsaicin analogs (capsinoids) found in non-pungent chili pepper (CH-19 Sweet) fruits. Biosci Biotechnol Biochem. 2010;74(2):274–278. doi: 10.1271/bbb.90570 20139632

[33] American College of Sports Medicine . ACSM’s guidelines for exercise testing and prescription. In: Liguori G 11th ed. Wolters Kluwer. 2021.

[34] Bishop D , Girard O , Mendez-Villanueva A . Repeated sprint ability. Sports Med. 2011;41(9):741–756. 10.2165/11590560-000000000-00000 21846163

[35] McArdle WD , Katch FI , Katch VL , et al. Exercise Physiology In: Nutrition, Energy, and Human Performance. 8 Philadelphia: Wolters Kluwer Health/Lippincott Williams & Wilkins; 2015 8th ed.

[36] Knuttgen HG , Kraemer WJ . Exercise terminology. J Appl Sport Sci Res. 1987;1(1):1–10.

[37] Cormie P , McGuigan MR , Newton RU . Neuromuscular power. Sports Med. 2011;41(1):17–38. 10.2165/11537690-000000000-00000 21142282

[38] Oliver JL , Ramachandran AK , Singh U , et al. The effects of strength, plyometric and combined training on strength, power and speed characteristics in high-level, highly trained Male youth soccer players: a systematic review and meta-analysis. Sports Med. 2024;54(3):623–643. doi: 10.1007/s40279-023-01944-8 PMC10978689 37897637

[39] Kraemer WJ , Ratamess NA . Fundamentals of resistance training: progression and exercise prescription. Med Sci Sports Exercise. 2004;36(4):674–688. doi: 10.1249/01.MSS.0000121945.36635.61 15064596

[40] Bassett DR , Howley ET . Limiting factors for maximum oxygen uptake and determinants of endurance performance. Med Sci Sports Exercise. 2000;32(1):70–84. doi: 10.1097/00005768-200001000-00012 10647532

[41] Poole DC , Jones AM . Oxygen uptake kinetics. Comprehensive Physiology. 2012;2:933–996. doi: 10.1002/cphy.c100072 23798293

[42] R Core Team . R: A language and environment for statistical computing [Computer software]. R Foundation for Statistical Computing; 2022. https://www.R-project.org/

[43] Wickham H . ggplot2. Wiley Interdiscip Rev Comput Stat. 2011;3:180–185. doi: 10.1002/wics.147

[44] Sterne JAC , Sutton AJ , Ioannidis JPA , et al. Recommendations for examining funnel plot asymmetry. Brit Med J. 2011;343:d4002. doi: 10.1136/bmj.d4002 21784880

[45] Peters JL , Sutton AJ , Jones DR , et al. Contour-enhanced funnel plots. J Clin Epidemiol. 2008;61(10):991–996. doi: 10.1016/j.jclinepi.2007.11.010 18538991

[46] Egger M , Davey Smith G , Schneider M , et al. Bias in meta-analysis detected by regression test. Brit Med J. 1997;315(7109):629–634. doi: 10.1136/bmj.315.7109.629 PMC2127453 9310563

[47] Viechtbauer W , Cheung MWL . Outlier and influence diagnostics for meta-analysis. Res Synth Methods. 2010;1(2):112–125. doi: 10.1002/jrsm.11 26061377

[48] Lauersen JB , Andersen TE , Andersen LB . Strength training as superior, dose-dependent and safe prevention of acute and overuse sports injuries: a systematic review, qualitative analysis and meta-analysis. Br J Sports Med. 2018;52(24):1557–1563. doi: 10.1136/bjsports-2018-099078 30131332

[49] GRADE Working Group . GRADE: an emerging consensus on rating quality of evidence. Brit Med J. 2008;336(7650):924–926. doi: 10.1136/BMJ.39489.470347.AD PMC2335261 18436948

[50] Egan B , Zierath JR . Exercise metabolism and the molecular regulation of skeletal muscle adaptation. Cell Metab. 2013;17(2):162–184. doi: 10.1016/j.cmet.2012.12.012 23395166

[51] Pickering C , Kiely J . Are the current guidelines on caffeine use in sport optimal for everyone? Inter-individual variation in caffeine ergogenicity, and a move towards personalised sports nutrition. Sports Med. 2018;48(1):7–16. doi: 10.1007/s40279-017-0776-1 PMC5752738 28853006

[52] Gabriel DA , Kamen G , Frost G . Neural adaptations to resistive exercise: mechanisms and recommendations for training practices. Sports Med. 2006;36(2):133–149. doi: 10.2165/00007256-200636020-00004 16464122

[53] Takahashi H , Inaki M , Fujimoto K , et al. Control of the rate of phosphocreatine resynthesis after exercise in trained and untrained human quadriceps muscles. Eur J Appl Physiol. 1995;71(5):396–400. doi: 10.1007/BF00240409 8565970

[54] Seol S-H , Chung G . Estrogen-dependent regulation of transient receptor potential vanilloid 1 (TRPV1) and P2X purinoceptor 3 (P2X3): implication in burning mouth syndrome. J Dent Sci. 2021;16(4):1234–1242. doi: 10.1016/j.jds.2021.06.007 PMC8739235 35028015

[55] Xu S , Cheng Y , Keast JR , et al. 17β-Estradiol activates estrogen receptor β-signalling and inhibits transient receptor potential vanilloid receptor 1 activation by capsaicin in adult rat nociceptor neurons. Endocrinology. 2008;149(11):5540–5548. doi: 10.1210/en.2008-0278 PMC2584594 18617618

[56] Fillingim RB , King CD , Ribeiro-Dasilva MC , et al. Sex, gender, and pain: a review of recent clinical and experimental findings. J Pain. 2009;10(5):447–485. doi: 10.1016/j.jpain.2009.01.001 PMC2677686 19411059

[57] Linthorne NP . Analysis of standing vertical jumps using a force platform. AmJPh. 2001;69(11):1198–1204. doi: 10.1119/1.1397460

[58] Sahlin K . Muscle energetics during explosive activities and potential effects of nutrition and training. Sports Med. 2014;44(Suppl 2):167–173. doi: 10.1007/s40279-014-0256-9 PMC4213384 25355190

[59] Venegas-Carro M , Herring JT , Riehle S , et al. Jumping vs. Running: effects of exercise modality on aerobic capacity and neuromuscular performance after a six-week high-intensity interval training. PLoS One. 2023;18(2):e0281737. doi: 10.1371/journal.pone.0281737 PMC9917273 36763697

[60] Fitts RH , McDonald KS , Schluter JM . Determinants of muscle force and power. J Biomech. 1991;24(Suppl 1):111–122. doi: 10.1016/0021-9290(91)90382-W 1791172

[61] Enoka RM , Duchateau J . Rate coding and muscle force. Cold Spring Harbor Perspect Med. 2017;7(10):a029702. doi: 10.1101/cshperspect.a029702 PMC5629984 28348173

[62] de Freitas MC , Cholewa JM , Freire RV , et al. Acute capsaicin supplementation improves 1500-m running time-trial performance and rate of perceived exertion in physically active adults. J Strength Cond Res. 2018;32(2):572–577. doi: 10.1519/JSC.0000000000002329 29120986

[63] Janssens PLHR , Hursel R , Westerterp-Plantenga MS . Capsaicin increases satiety. PLoS One. 2013;8(11):e7786. doi: 10.1371/journal.pone.0077786 PMC3699483 23844093

[64] Oh JY , Ohta H . Capsaicin reduces fat in rats. J Nutr Sci Vitaminol. 2003;49(2):107–113. doi: 10.3177/jnsv.49.107 12887156

[65] Joyner MJ , Coyle EF . Endurance exercise performance: the physiology of champions. J Physiol. 2008;586(1):35–44. doi: 10.1113/jphysiol.2007.143834 PMC2375555 17901124

[66] Rollyson WD , Stover CA , Brown KC , et al. Capsaicin bioavailability. J Control Release. 2014;196:96–105. doi: 10.1016/j.jconrel.2014.09.027 PMC4267963 25307998

[67] European Food Safety Authority (EFSA) Panel on Food Additives and Flavourings . Safety of phenylcapsaicin as a novel food pursuant to regulation (EU) 2015/2283. EFSA J. 2019;17(6):5718. doi: 10.2903/j.efsa.2019.5718 PMC7009045 32626340

[68] Baskaran P , Krishnan V , Ren J . Capsaicin-induced effects on metabolic rate and energy expenditure. Molecules. 2018;23(12):3198. doi: 10.3390/molecules23123198

[69] Ohnuki K , Haramizu S , Oki K , et al. Capsiate promotes energy metabolism. Am J Physiol Regul Integr Comp Physiol. 2001;281(6):R1603–R1609. doi: 10.1152/ajpregu.2001.281.6.R1603

[70] Snitker S , Fujishima Y , Shen H , et al. Capsinoids and energy metabolism. AJCN. 2009;89(1):45–50. doi: 10.3945/ajcn.2008.26561 PMC3151435 19056576

[71] Jiménez-Martínez P , Alix-Fages C , Jurado-Fasoli L , et al. Capsaicinoids and capsinoids in exercise performance: systematic review. Biol Sport. 2022;39(4):919–930. doi: 10.5114/biolsport.2022.109455

[72] de Freitas MC , Billaut F , Panissa VLG , et al. Capsaicin supplementation increases time to exhaustion in high-intensity intermittent exercise without modifying metabolic responses in physically active men. Eur J Appl Physiol. 2019;119(4):971–979. doi: 10.1007/s00421-019-04086-w 30737555

[73] Giuriato G , Giuriato G , Venturelli M , et al. Capsaicin effects on performance, fatigue and inflammation. Nutrients. 2022;14(2):232. doi: 10.3390/nu14020232 PMC8778706 35057413

[74] Opheim MN . Effect of capsaicin supplementation on repeated sprinting. Master’s thesis. Virginia Tech; 2010. https://vtechworks.lib.vt.edu/handle/10919/33589 10.1519/JSC.0b013e3182429ae5 22130402

[75] de Oliveira EP , Burini RC , Jeukendrup A . Gastrointestinal complaints during exercise: prevalence, etiology, and nutritional recommendations. Sports Med. 2014;44(Suppl 1):79–85. doi: 10.1007/s40279-014-0153-2 PMC4008808 24791919

[76] Fischer MJM , Ciotu CI , Szallasi A . Capsaicin-sensitive afferents. Front Physiol. 2020;11:554195. doi: 10.3389/fphys.2020.554195 PMC7772409 33391007

[77] Pickering C , Kiely J . Blinding problem in sport science research. Sports Med. 2019;49(12):1943–1951. doi: 10.1007/s40279-019-01186-w

[78] Grgic J , Mikulic P , Rodriguez-Giustiniani G . Capsaicin effects on performance and perceived effort. Nutrients. 2022;14(7):1471. doi: 10.3390/nu14071471

[79] Parra-Vargas M , Rico-Sanz J . Mechanisms of capsaicin in endurance performance. Front Physiol. 2022;13:852504. doi: 10.3389/fphys.2022.852504

[80] Moura HP , de Oliveira MR , de Freitas MC . Capsaicin supplementation critical review. Int J Sport Nutr Exerc Metab. 2021;31(4):361–370. doi: 10.1123/ijsnem.2020-0252

[81] de Freitas MC , Gerosa-Neto J , Zanchi NE , et al. Epinephrine, lactate and SBP responses to capsaicin. J Sci Med Sport. 2018;21(12):1213–1218. doi: 10.1016/j.jsams.2018.05.011

