# Supplementary material for: Effects of acute Capsaicin analogue intake on athletic performance: a three-level meta-analysis
Source: J Int Soc Sports Nutr. 2026 Sep 30;23(1):2733576. doi: 10.1080/15502783.2026.2733576 (PMC13629781; doi:10.1080/15502783.2026.2733576)
Supplement: Supplementary file 1 [file RSSN_A_2733576_SM4801.docx]

**Electronic Supplementary Material Contents**

| **Number** | **Material** | **Page** |
| --- | --- | --- |
| 1 | Electronic Supplementary Material Appendix S1 (PRISMA 2020 checklist) | P2-5 |
| 2 | Electronic Supplementary Materials Appendix S2 (Basic characteristics table of documents) | P6-23 |
| 3 | Electronic Supplementary Material Appendix S3 (Funnel Plot) | P24-26 |
| 4 | Electronic Supplementary Material Appendix S4 (Power Visualization) | P27-28 |
| 5 | Electronic Supplementary Material Appendix S5 (PEDro Assessment) | P29-31 |
| 6 | Electronic Supplementary Material Appendix S6 (GRADE Assessment) | P31-34 |
| 7 | Electronic Supplementary Material Appendix S7 (A Sensitivity Analysis based on level 2 and level 3 Leave-one-out) | P35-39 |

**Electronic Supplementary Material Appendix S1**

| **Section and Topic** | **Item #** | **Checklist item** | **Location where item is reported (on page)** |
| --- | --- | --- | --- |
| **TITLE** | | |  |
| Title | 1 | Identify the report as a systematic review. | P1 |
| **ABSTRACT** | | |  |
| Abstract | 2 | See the PRISMA 2020 for Abstracts checklist. | P1-2 |
| **INTRODUCTION** | | |  |
| Rationale | 3 | Describe the rationale for the review in the context of existing knowledge. | P2 |
| Objectives | 4 | Provide an explicit statement of the objective(s) or question(s) the review addresses. | P3 |
| **METHODS** | | |  |
| Eligibility criteria | 5 | Specify the inclusion and exclusion criteria for the review and how studies were grouped for the syntheses. | P4 |
| Information sources | 6 | Specify all databases, registers, websites, organisations, reference lists and other sources searched or consulted to identify studies. Specify the date when each source was last searched or consulted. | P4 |
| Search strategy | 7 | Present the full search strategies for all databases, registers and websites, including any filters and limits used. | P5 |
| Selection process | 8 | Specify the methods used to decide whether a study met the inclusion criteria of the review, including how many reviewers screened each record and each report retrieved, whether they worked independently, and if applicable, details of automation tools used in the process. | P5 |
| Data collection process | 9 | Specify the methods used to collect data from reports, including how many reviewers collected data from each report, whether they worked independently, any processes for obtaining or confirming data from study investigators, and if applicable, details of automation tools used in the process. | P5 |
| Data items | 10a | List and define all outcomes for which data were sought. Specify whether all results that were compatible with each outcome domain in each study were sought (e.g. for all measures, time points, analyses), and if not, the methods used to decide which results to collect. | P6 |
|  | 10b | List and define all other variables for which data were sought (e.g. participant and intervention characteristics, funding sources). Describe any assumptions made about any missing or unclear information. | P6 |
| Study risk of bias assessment | 11 | Specify the methods used to assess risk of bias in the included studies, including details of the tool(s) used, how many reviewers assessed each study and whether they worked independently, and if applicable, details of automation tools used in the process. | P6-7 |
| Effect measures | 12 | Specify for each outcome the effect measure(s) (e.g. risk ratio, mean difference) used in the synthesis or presentation of results. | P7 |
| Synthesis methods | 13a | Describe the processes used to decide which studies were eligible for each synthesis (e.g. tabulating the study intervention characteristics and comparing against the planned groups for each synthesis (item #5)). | P7-8 |
|  | 13b | Describe any methods required to prepare the data for presentation or synthesis, such as handling of missing summary statistics, or data conversions. | P8-9 |
|  | 13c | Describe any methods used to tabulate or visually display results of individual studies and syntheses. | P14-18 |
|  | 13d | Describe any methods used to synthesize results and provide a rationale for the choice(s). If meta-analysis was performed, describe the model(s), method(s) to identify the presence and extent of statistical heterogeneity, and software package(s) used. | P9-11 |
|  | 13e | Describe any methods used to explore possible causes of heterogeneity among study results (e.g. subgroup analysis, meta-regression). | P11 |
|  | 13f | Describe any sensitivity analyses conducted to assess robustness of the synthesized results. | P12 |
| Reporting bias assessment | 14 | Describe any methods used to assess risk of bias due to missing results in a synthesis (arising from reporting biases). | P11-12 |
| Certainty assessment | 15 | Describe any methods used to assess certainty (or confidence) in the body of evidence for an outcome. | P12 |
| **RESULTS** | | |  |
| Study selection | 16a | Describe the results of the search and selection process, from the number of records identified in the search to the number of studies included in the review, ideally using a flow diagram. | P12-13 |
|  | 16b | Cite studies that might appear to meet the inclusion criteria, but which were excluded, and explain why they were excluded. | P13 |
| Study characteristics | 17 | Cite each included study and present its characteristics. | P13-14 |
| Risk of bias in studies | 18 | Present assessments of risk of bias for each included study. | P17-18 |
| Results of individual studies | 19 | For all outcomes, present, for each study: (a) summary statistics for each group (where appropriate) and (b) an effect estimate and its precision (e.g. confidence/credible interval), ideally using structured tables or plots. | P14-16 |
| Results of syntheses | 20a | For each synthesis, briefly summarise the characteristics and risk of bias among contributing studies. | P14 |
|  | 20b | Present results of all statistical syntheses conducted. If meta-analysis was done, present for each the summary estimate and its precision (e.g. confidence/credible interval) and measures of statistical heterogeneity. If comparing groups, describe the direction of the effect. | P14,18 |
|  | 20c | Present results of all investigations of possible causes of heterogeneity among study results. | P14-18 |
|  | 20d | Present results of all sensitivity analyses conducted to assess the robustness of the synthesized results. | P18 |
| Reporting biases | 21 | Present assessments of risk of bias due to missing results (arising from reporting biases) for each synthesis assessed. | P17-18 |
| Certainty of evidence | 22 | Present assessments of certainty (or confidence) in the body of evidence for each outcome assessed. | P18 |
| **DISCUSSION** | | |  |
| Discussion | 23a | Provide a general interpretation of the results in the context of other evidence. | P19-23 |
|  | 23b | Discuss any limitations of the evidence included in the review. | P23-24 |
|  | 23c | Discuss any limitations of the review processes used. | P23-24 |
|  | 23d | Discuss implications of the results for practice, policy, and future research. | P22-24 |
| **OTHER INFORMATION** | | |  |
| Registration and protocol | 24a | Provide registration information for the review, including register name and registration number, or state that the review was not registered. | P3 |
|  | 24b | Indicate where the review protocol can be accessed, or state that a protocol was not prepared. | P3 |
|  | 24c | Describe and explain any amendments to information provided at registration or in the protocol. | N/A |
| Support | 25 | Describe sources of financial or non-financial support for the review, and the role of the funders or sponsors in the review. | P27 |
| Competing interests | 26 | Declare any competing interests of review authors. | P28 |
| Availability of data, code and other materials | 27 | Report which of the following are publicly available and where they can be found: template data collection forms; data extracted from included studies; data used for all analyses; analytic code; any other materials used in the review. | N/A |

**Electronic Supplementary Materials Appendix S2 (Basic characteristics table of documents)**

| Study;  Study design | Exercise protocol | Sample;  Training status | Mean age (y) and body weight (kg) | Dosage of CAP and PLA;  Form;  Intake timepoint | Washout period | Performance outcomes | Statistical significance | |
| --- | --- | --- | --- | --- | --- | --- | --- | --- |
| Costa et al.(2020);  RDB  Crossover | 400 m or 3000 m running | 12 males;  Physically active | 28.6±5.4/  175.0 ± 5.4 | CAP:2 * 6 mg capsaicin  PLA: 12 mg starch；  Capsule;  45 minutes before | 1 week | 400 m time (s):  CAP: 66.4±4.2 vs PLA: 67.1±4.8  3000 m time (s): CAP:893.9±46.8 vs PLA:915.2±67.6； | YES  (p < 0.05) |  |
| Cross et al.(2020)  RSB  Crossover | Twice isokinetic knee extensor contraction function test | 4 males  5 females;  Recreationally active | 23.6 ± 1.5/  24.2 ± 3.3 | CAP:1.2 mg capsaicin  PLA:1.2 mg;  Candy;  45 minutes before | 3-14 days | Peak torque (N⋅m⁻¹):CAP: 126.0 ± 40.4 vs PLA:118.8 ± 41.3;  Total torqu (N⋅m⁻¹):CAP: 8012 ± 2771 vs PLA: 7823 ± 2611 | YES  Only with peak torque  (p < 0.05) |  |
| da Cruz et al.(2023)  RDB  Crossover | 70% 1RM bench press TTE;  70% 1RM incline bench press TTE | 14 males;  Experience in RT | 24.8 ± 3.3/  82.4 ± 7.1 | CAP:12 mg capsaicin  PLA:6 * 50 mg Starch;  Capsule;  45 minutes before | 1 week | MNR (reps):  CAP: 55.0 ± 10.0 vs PLA: 54.5 ± 8.4;  Total volume (kg):  CAP: 7332.7 ± 1866.2 vs PLA: 7257.0 ± 1644.6; | NO  (P > 0.05) |  |
| da Silva et al.(2022);  RDB  Crossover | 4 * 60% 1RM FBP | 12 males;  Jiu-jitsu athlete | 24.3 ± 1.5/  75.7 ± 10.1 | CAP:12 mg capsaicin  PLA:12 mg Celulomax E;  Capsules;  45 minutes before | 1 week | MPO(w) CAP: 527 ± 85 vs PLA: 504 ± 86  PV (m/s)  CAP: 1.40 ± 0.11 vs PLA: 1.34 ± 0.12 | YES  (p = 0.001) |  |
| de Freitas et al.(2022)  RDB  Crossover | 5-kilometer running and 4 * 70% 1RM half-squat TTE | 11males;  Resistance-trained; | 176.3 ± 5.3/  79.8 ± 11 | CAP:2 * 12 mg capsaicin  PLA:2 * 12 mg starch;  Capsules;  45 minutes before and 12 mg Immediately 12mg | 1 week | Peak heart rate (b·min^−1^):  CAP 178.7 ± 10.9 vs PLA: 183.1 ± 7.7  Average heart rate (b·min^−1^) :  CAP: 153.4 ± 12.9 vs PLA: 158.0 ± 12.3  Total volume (kg): | YES  (P < 0.05) |  |
|  |  |  |  |  |  | CAP: 2077.6 ± 465.2 vs PLA:1838.9 ± 624.1  Number of repetitions(kg):  CAP: 34.9 ± 8.2 vs PLA:30.5 ± 10.2 |  |  |
| de Freitas et al. (2019)  RDB  Crossover | HIIT | 13 males;  Physically active | 24.4±4.0/  78.7±13.8 | CAP:12 mg capsaicin  PLA:12 mg starch;  Capsule;  45 minutes before | 1 week | TTE(s):CAP:1530 ± 515 vs PLA: 1342 ± 446  V̇O_2_ average  (mL/kg/min):  CAP: 38.1±6.9 vs PLA: | YES  Only with TTE  (P < 0.05) |  |
|  |  |  |  |  |  | 39.4±7.7  Time to reach 90% of V̇O_2Peak_(s): |  |  |
|  |  |  |  |  |  | CAP: 266±204 vs PLA: 165±138 |  |  |
| de Freitas et al.  (2018)  RDB  Crossover | 4 * 70% 1 RM squat TTE | 10 males;  Resistance-trained | 22.7 ± 4.0/  82.3 ± 9.6 | CAP:12 mg capsaicin  PLA:50 mg starch  Capsule;  45 minutes before | 1 week | Total weight lifted in weightlifting (kg):  CAP: 3179.6 ± 942.4 vs PLA: 3179.6 ± 942.4  Maximum repetition count(reps):  CAP: 42.6 ± 12.6 vs PLA: 35.1 ± 12.3 | YES  (p < 0.05) |  |
| de Freitas et al.  (2018)  RDB  Crossove | 1500 m running | 10 males;  Physically active | 23.5±1.9/  78.3±12.4 | CAP:12 mg capsaicin  PLA:50 mg starch;  Capsule;  45 minutes before | 1 week | Time (s):  CAP: 371.6 ± 40.8 vs PLA: 376.7 ± 39 | YES  (p = 0.009) |  |
| de Moura et al.(2022)  RDB  Crossover | 70% 1 RM bench pressd TTE | 20 males;  Resistance-trained | 28 ± 4.1/  176.0 ± 6.4 | CAP:6 mg or 12 mg capsiate  PLA: 6 mg or 12 mg starch;  Capsule;  45 minutes before | 3 days | Total weight lifted of 6 mg,12 mg:  CAP(6mg): 2454.6 ± 448.6 vs PLA: 2354.7 ± 458.6 vs CAP (12mg): 2309.3 ± 428.1 | YES  (p < 0.05)  Only with 6 mg |  |
| Dos Santos Gomes et al.(2022)  RDB  Crossover | MVIC | 13 males;  Strength trained | 25.2±3.2/  179±10 | CAP:2 * 6 mg capsiate  PLA:2 * 6 mg starch;  Capsule;  45 minutes before | 1 week | Peak force（N）of  MVIC1, MVIC2, MVIC3, MVIC4, MVIC5:CAP: 697.11 ± 114.49 vs PLA: 687.12 ± 107.33, 600.02 ± 110.94 vs 576.79 ± 91.15, 592.45 ± 106.15 vs 559.82 ± 95.22, 580.09 ± 101.11 vs PLA: 545.13 ± 85.81, 569.73 ± 96.54 vs 530.33 ± 86.73  Mean force（N）of  MVIC1, MVIC2, MVIC3, MVIC4, MVIC5:CAP: 594.79 ± 87.85 vs PLA: 574.10 ± 85.36, 524.61 ± 103.75 vs 501.64 ± 81.52, 506.78 ± 88.06 vs 490.23 ± 92.70, 497.62 ± 91.94 vs 475.44 ± 84.71, 483.24 ± 97.78 vs 460.80 ± 85.96  Minimum force (N) of MVIC1, MVIC2, MVIC3, MVIC4, MVIC5:CAP: 449.67 ± 128.07 vs PLA: 481.44 ± 85.76, 448.37 ± 156.84 vs 424.89 ± 76.16, 418.89 ± 90.38 vs 388.68 ± 144.51, 356.24 ± 136.18 vs 380.17 ± 98.44, 385.33 ± 141.94 vs 374.91 ± 80.20 | YES  (P < 0.05) |  |
| Fonseca et al.(2023)  RDB  Crossover | 1RM test protocol | 14 males  Resistance-trained | 22.21 ± 2.55/  82.35 ±5.16 | CAP:12 mg Capsaicin  PLA:20 mg starch;  Capsule;  45 minutes before | 1 week | NMR (reps):  CAP: 92.00 ± 23.32 vs PLA: 98.85 ± 24.27  VL (kg):CAP: 16,225 ± 5,033 vs PLA: 18,254 ± 5,749 | NO  (P > 0.05) |  |
| Jiménez-Martínez et al. (2023)  RTB  Crossover | 2 * 60% 1RM SQ,  3 * 8 of 70% 1RM SQ,2 * 60% 1RM SQ; | 25 males  Healthy | 21.0±2.2/  76.5±9.5 | CAP: 0.625 mg or 2.5 mg phenylcapsaicin; PLA:0.625 mg or 2.5 mg maltodextrin;  Capsule;  45 minutes before | 1 week | Maximum speed (m·s ^−1^) of LD,HD, PLA:0.78 ± 0.03, 0.80 ± 0.06, 0.78 ± 0.04  Average speed (m·s ^−1^) of LD, HD, PLA: 0.61 ± 0.07, 0.64 ± 0.07, 0.59 ± 0.05  Slowest speed (m·s ^−1^) of LD, HD, PLA: 0.45 ± 0.09, 0.49 ± 0.09, 0.43 ± 0.10 | YES  (P < 0.05)  Only with SQ |  |
| Jiménez-Martínez et al.(2023)  RTB  Crossover | 3 × 8 × 70% 1RM squat,  CMJ,  60% 1RM squat | 25 males  Resistance-trained | 21.0±2.2 /  76.5±9.5 | CAP: 0.625 mg or 2.5 mg phenylcapsaicin; PLA:0.625 mg or 2.5 mg maltodextrin;  Capsule;  45 minutes before | 1 week | Total volume load(kg):LD,HD,PLA: 2014.6 ± 380.9, 2101.7 ± 352.9, 2089.1 ± 357.6  Pre CMJ height(cm):LD, HD, PLA: 40.15 ± 9.61, 40.18 ± 9.88, 39.94 ± 9.63;  Post CMJ height(cm):LD,HD,PLA: 33.13 ± 8.29, 34.73 ± 9.08, 32.92 ± 8.24 | YES  (P < 0.05) |  |
| Langan et al.(2020)  RSB  Crossover | Power bike TTE | 8 males  5 females  Recreationally active | Males: 25.3 ± 3.1/ 179.9 ± 3.6;  Females: 22.3 ± 2.1/ 162.8 ± 9.0 | CAP:1.2 mg capsaicin  PLA: Fruit soft candy;  Candy;  45 minutes before | 1 week | Time to exhaustion (s):  CAP: 517.5 ± 258.4 vs PLA: 487.8 ± 187.7 | NO  (P ≥ 0.05) |  |
| Oliveira et al.(2023)  RDB  Crossover | CrossFit® | 17 males  Resistance-trained | 27.3 ± 3.6/  79.1 ± 8.2 | CAP:2 * 6 mg capsiate  PLA:2 * 6 mg starch;  Capsule;  45 minutes before | 1 week | Number of repetitions(n):  CAP of 12mg,24mg vs PLA: (12 mg) 627.7 ± 99.8, (24 mg) 631.3 ± 120.1 vs PLA: 620.5 ± 137.8; | NO  (P = 0.768) |  |
| Osuna-Prieto et al.(2023)  RTB  Crossover | CMJ,BPT,  1 RM squat,  1 RM bench | 19 males  6 females  Resistance training experience | 23.0 ± 3.7/  76.6 ± 6.3 | CAP:4 * 3 mg dihydrocapsiate  PLA:250 mg maltodextrin;  Capsule;  30 minutes before | 1 week | Height (cm);  CAP:33.20 ± 8.07 vs PLA:33.32 ± 7.85  Speed (m/s)  CAP:2.82 ± 0.77  vs PLA:2.82 ± 0.74  1 RM squat weight (kg):  CAP:123.76 ± 40.63 vs PLA:122.66 ± 40.97  1 RM bench press weight (kg):  CAP:99.47 ± 43.09 vs PLA:99.60 ± 43.34 | NO  (P ≥ 0.05) |  |
| Padilha et al.(2020)  RDB  Crossover | 90% s V̇O₂peak running TTE | 15 males  Leisure training | 22.1 ± 3.0/  179.0 ± 0.6 | CAP:12 mg capsaicin  PLA:12 mg;  Capsule;  45 minutes before | 1 week | V̇O_₂peak_ (s)  CAP: 391.71±221.8 vs PLA: 298.20±174.5  Peak Lactate(µmol):CAP: 7.98±2.11 vs PLA: 8.58±2.15  TTE (s):  CAP:654.28±195.44 vs | NO  (P > 0.05) |  |
|  |  |  |  |  |  | PLA:709.20±208.44 |  |  |
| Simões et al.(2022)  RDB  Crossover  Triviño, A.R et al.(2026)  RTB  Crossover | 4 * 70% 1RM squat TTE  70% 1RM SQ  CMJ | 11 males  Resistance-trained  25 males  25 females  CrossFit-trained athletes | 30.2 ± 3.7/  75.0 ± 11.5 | CAP:2 * 12 mg  capsaicin  PLA:2 * 12 mg Starch;  Capsule;  45 minutes before  CAP:2.5 mg phenylcapsaicin  PLA:600 mg maltodextrin;  Capsule;  45 minutes before | 1 week  1 week | Total exercise volume (kg):  CAP:6010.0 ± 1067.0 vs PLA: 5505.2 ± 810.7  RPE:CAP: 15.41 ± 2.9 vs PLA: 15.43 ± 3.04  CMJ (height):  CAP: −1.26 ± 0.46 vs PLA: −2.25 ± 0.47;  70% 1RM SQ (kg):  CAP: 78.5 ± SD vs PLA: 76.4 ± SD  70% 1RM (n)：  CAP: 22.3 ± SD vs PLA: 20.7 ± SD | NO  (P > 0.05) |  |
| Volino-Souza et al.(2023)  RDB  Crossover | Leg extension: 3 × 70% 1RM | 8 males  Recreational activity | 33.33±7.42/  75.38±12.63 | CAP:12 mg capsaicin  PLA:250 mg maltodextrin;  Capsule;  45 minutes before | 1 week | Number of repetitions(reps): CAP of set 1,set 2,set 3: 15±5, 11±3, 9±2 vs PLA of set 1,set 2,set 3: 15±2，10±3，8±2  Time of exercise (s)：CAP of set 1, set 2, set 3: 42.70±17.72, 18.65±6.21, 16.40±5.57 vs PLA of set 1, set 2, set 3: 49.74±14.40, 17.80±6.00, 15.45±5.72  Blood Lactate (mmol·L^−1^): CAP of set 1, set 2, set 3: 37.53±11.89, 194.67±54.20, 105.15±36.26 vs PLA of set 1, set 2, set3:26.13±8.37, 157.12±62.84, 96.22±22.04 | NO  (P > 0.05) |  |
| von Ah Morano et al.(2021)  RDB  Crossover | 10 km running | 21 males  Amateur athletes | 29.3±5.5/  74.2±11.3 | CAP:2 * 12 mg  PLA:2 * 12 mg ;  Capsule;  45 minutes before | 1 week | Time (min):CAP: 45.07 ± 6.41 vs PLA: 45.13 ± 6.73 | NO  (p = 0.828) |  |

**Electronic Supplementary Material Appendix S3 (Funnel Plot)**

*Note: Colored circles represent different effect size points. A is the funnel chart before the subtraction method, and B is the funnel chart after the subtraction method.*

***Note:*** *Colored circles represent different effect sizes. From A to L, they represent male, mixed sex, double-blind, triple-blind, trained, capcasin, capsiate, phenylcapsaicin, aerobic capacity, muscular endurance, power, and strength, respectively.*

**Electronic Supplementary Material Appendix S4 (Power Visualization)**

**Electronic Supplementary Material Appendix S5 (PEDro Assessment)**

| **Study** | **Item 1** | **Item 2** | **Item 3** | **Item 4** | **Item 5** | **Item 6** | **Item 7** | **Item 8** | **Item 9** | **Item 10** | **Item 11** | **Total** |
| --- | --- | --- | --- | --- | --- | --- | --- | --- | --- | --- | --- | --- |
|  |  |  |  |  |  |  |  |  |  |  |  |  |
| Costa et al. (2020) | 1 | 1 | 0 | 1 | 1 | 1 | 0 | 1 | 1 | 1 | 1 | 8 |
| Cross et al. (2020) | 1 | 1 | 0 | 1 | 1 | 0 | 0 | 1 | 1 | 1 | 1 | 7 |
| Cruz et al. (2023) | 1 | 1 | 0 | 1 | 1 | 1 | 0 | 1 | 1 | 1 | 1 | 8 |
| Silva et al. (2022) | 1 | 1 | 1 | 1 | 1 | 1 | 0 | 1 | 1 | 1 | 1 | 9 |
| Freitas et al. (2022) | 1 | 0 | 1 | 1 | 1 | 1 | 0 | 1 | 1 | 1 | 1 | 8 |
| Freitas et al. (2019) | 1 | 0 | 1 | 1 | 1 | 1 | 0 | 1 | 1 | 1 | 1 | 9 |
| 1. Freitas et al. (2018) | 1 | 1 | 1 | 1 | 1 | 1 | 0 | 1 | 1 | 1 | 1 | 9 |
| 2. Freitas et al. (2018) | 1 | 1 | 1 | 1 | 1 | 1 | 0 | 1 | 1 | 1 | 1 | 9 |
| Moura et al. (2022) | 1 | 1 | 1 | 1 | 1 | 1 | 0 | 0 | 1 | 1 | 1 | 9 |
| Gomes et al. (2022) | 1 | 1 | 1 | 1 | 1 | 1 | 0 | 1 | 1 | 1 | 1 | 9 |
| Fonseca et al. (2023) | 1 | 1 | 0 | 1 | 1 | 1 | 0 | 1 | 1 | 1 | 1 | 8 |
| 1. Jiménez-Martínez et al. (2023) | 1 | 1 | 1 | 1 | 1 | 1 | 1 | 1 | 1 | 1 | 1 | 10 |
| 2. Jiménez-Martínez et al. (2023) | 1 | 1 | 1 | 1 | 1 | 1 | 1 | 1 | 1 | 1 | 1 | 10 |
| Langan et al. (2020) | 1 | 1 | 0 | 1 | 0 | 0 | 0 | 1 | 1 | 1 | 1 | 6 |
| Oliveira et al. (2023) | 1 | 1 | 1 | 1 | 1 | 1 | 0 | 0 | 1 | 1 | 1 | 8 |
| Osuna-Prieto et al. (2023) | 1 | 1 | 1 | 1 | 1 | 1 | 1 | 0 | 1 | 1 | 1 | 9 |
| Padilha et al. (2020) | 1 | 1 | 1 | 1 | 1 | 1 | 0 | 1 | 1 | 1 | 1 | 9 |
| Simões et al. (2022) | 1 | 1 | 1 | 1 | 1 | 1 | 0 | 1 | 1 | 1 | 1 | 9 |
| Triviño, A.R et al. (2026) | 1 | 1 | 1 | 1 | 1 | 1 | 1 | 1 | 1 | 1 | 1 | 10 |
| Volino-Souza et al. (2023) | 1 | 1 | 0 | 1 | 1 | 1 | 0 | 1 | 1 | 1 | 1 | 8 |
| Morano et al. (2021) | 1 | 1 | 1 | 1 | 1 | 0 | 0 | 1 | 0 | 1 | 1 | 8 |

**Electronic Supplementary Material Appendix S6 (GRADE Assessment)**

| **Outcome** | **K** | **Certainty of Evidence Assessment** | | | | | **Hedge’s g [95% CI] *** | **GRADE†** |
| --- | --- | --- | --- | --- | --- | --- | --- | --- |
|  |  | **Risk of Bias** | **Inconsistency** | **Indirectness** | **Imprecision** | **Others** |  |  |
| Primary Outcome | | | | | | | | |
| Exercise performance | K = 67 | Serious | Serious | Not serious | Serious | Publication bias | 0.20 [0.07, 0.33] | ⨁◯◯◯ Very low |
| CAP Type | | | | | | | | |
| capsaicin | K = 30 | Serious | Not serious | Not serious | Not serious | None | 0.17 [0.00, 0.35] | ⨁⨁⨁◯ Moderate |
| capsiate | K = 18 | Serious | Serious | Not serious | Serious | None | 0.10 [-0.22, 0.42] | ⨁◯◯◯ Very low |
| phenylcapsaicin | K = 15 | Serious | Not serious | Not serious | Serious | None | 0.43 [0.15, 0.71] | ⨁⨁◯◯ Low |
| Gender | | | | | | | | |
| Male | K = 10 | Serious | Not serious | Not serious | Serious | Publication bias | 0.17 [0.02, 0.31] | ⨁⨁◯◯ Low |
| Mix | K = 57 | Serious | Very serious | Not serious | Not serious | None | 0.36 [0.07, 0.65] | ⨁⨁◯◯ Low |
| Training Level | | | | | | | | |
| Trained | K = 63 | Serious | Serious | Not serious | Not serious | Publication bias | 0.21 [0.07, 0.35] | ⨁⨁◯◯ Low |
| Untrained | K = 4 | Serious | Not serious | Not serious | Very serious | None | 0.17 [-0.26, 0.60] | ⨁◯◯◯ Very low |
| Exercise Type | | | | | | | | |
| Aerobic endurance | K = 10 | Serious | Not serious | Not serious | Not serious | None | 0.14 [-0.14, 0.42] | ⨁⨁⨁◯ Moderate |
| Muscular Endurance | K = 19 | Serious | Serious | Not serious | Serious | None | 0.25 [0.04, 0.47] | ⨁◯◯◯ Very low |
| Strength | K = 25 | Serious | Not serious | Not serious | Serious | None | 0.01 [-0.15, 0.30] | ⨁⨁◯◯ Low |
| Power | K = 13 | Serious | Not serious | Not serious | Serious | None | 0.07 [0.13, 0.64] | ⨁⨁◯◯ Low |
| Study Design | | | | | | | | |
| Triple Blind | K = 19 | Serious | Serious | Not serious | Serious | Publication bias | 0.33 [0.08, 0.58] | ⨁◯◯◯ Very low |
| Double Blind | K = 47 | Serious | Not serious | Not serious | Not serious | Publication bias | 0.16 [0.00, 0.31] | ⨁⨁◯◯ Low |
| ***Notes: K*:** the total number of effects included in the pooled effect size; ***Publication bias***, represented by Egger test p < 0.05.  ***:** The effect size (*Hedges’g*) was significant (p < 0.05).  **†** **GRADE Criteria for Certainty of Evidence**：  **High**: Very confident in the estimated effect.  **Moderate**: Moderately confident in the estimated effect.  **Low**: Limited confidence in the estimated effect.  **Very low**: Very limited confidence in the estimated effect | | | | | | | | |

**Electronic Supplementary Material Appendix S7 (A Sensitivity Analysis based on level 2 and level 3 Leave-one-out)**

**Note**: Each point represents the pooled Hedges’ g after removing one study at a time. Horizontal lines represent the corresponding 95% confidence intervals. I² represents residual heterogeneity estimated within the three-level model. Level 1 refers to sampling variance, Level 2 to within-study variance among effect sizes from the same study, and Level 3 to between-study variance. The analysis was conducted to assess whether the main pooled estimate was disproportionately influenced by any individual study.

***Note:*** *From A to L, they represent male, mixed sex, double-blind, triple-blind, trained, capcasin, capsiate, phenylcapsaicin, aerobic capacity, muscular endurance, power, and strength, respectively.*
